# Supplementary figures and images for: Genomic ascertainment to quantify prevalence and cancer risk in adults with pathogenic and likely pathogenic germline variants in RASopathy genes
Source: medRxiv. 2024 Oct 11:2024.10.09.24314324. Preprint. [Version 1] doi: 10.1101/2024.10.09.24314324 (PMC11722494; doi:10.1101/2024.10.09.24314324)

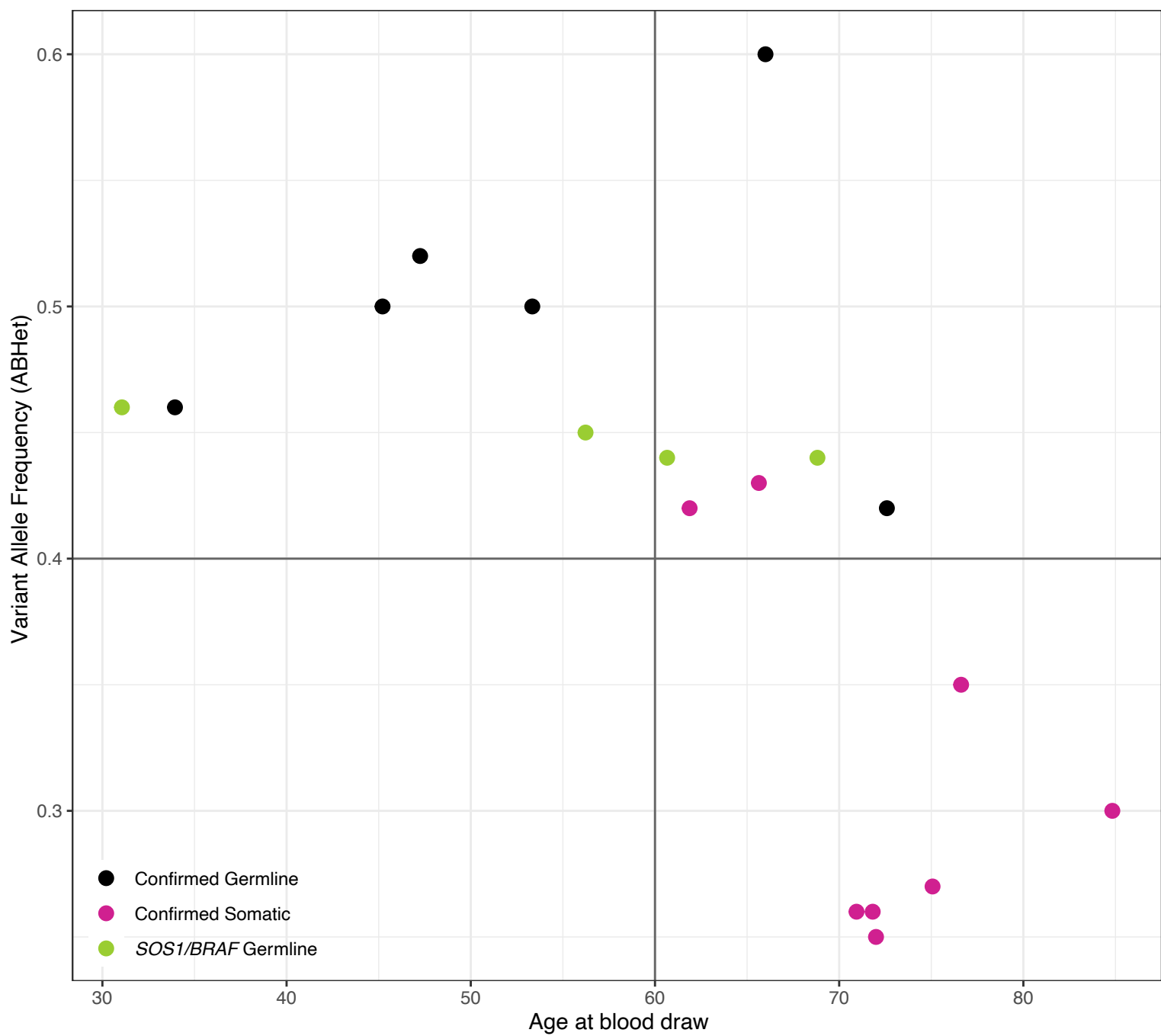

Supplement: Supplement 2 — Supplementary Figure 1. Plot of age at blood draw and variant allele frequency (VAF) of 16 variants in 18 individuals from Geisinger for whom a histologically benign tissue was available for Sanger sequencing. [file media-2.pdf]

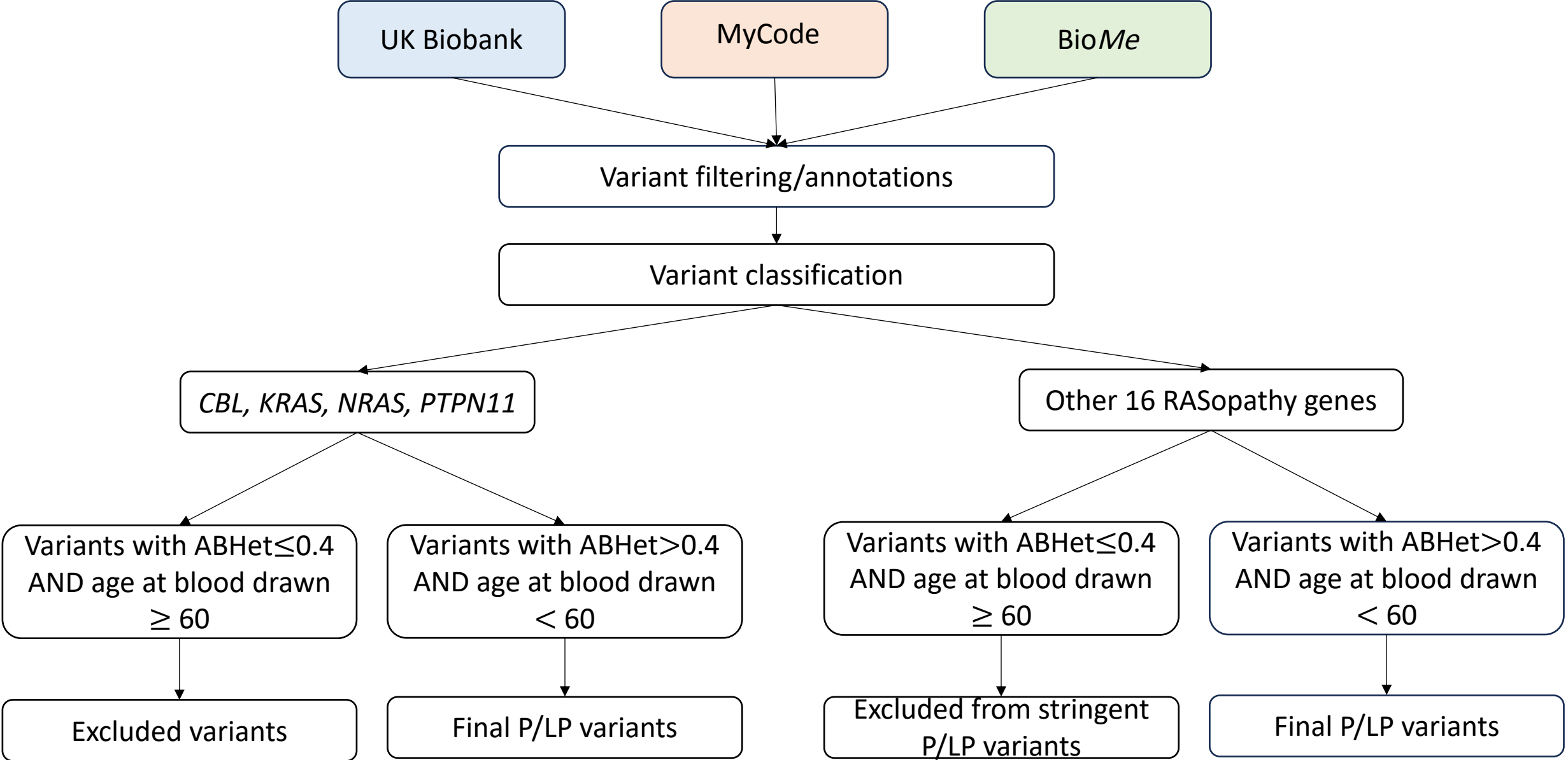

Supplement: Supplement 3 — Supplementary Figure 2. Flow diagram of approach to minimize the effects of clonal hematopoiesis in this study. [file media-3.pdf]

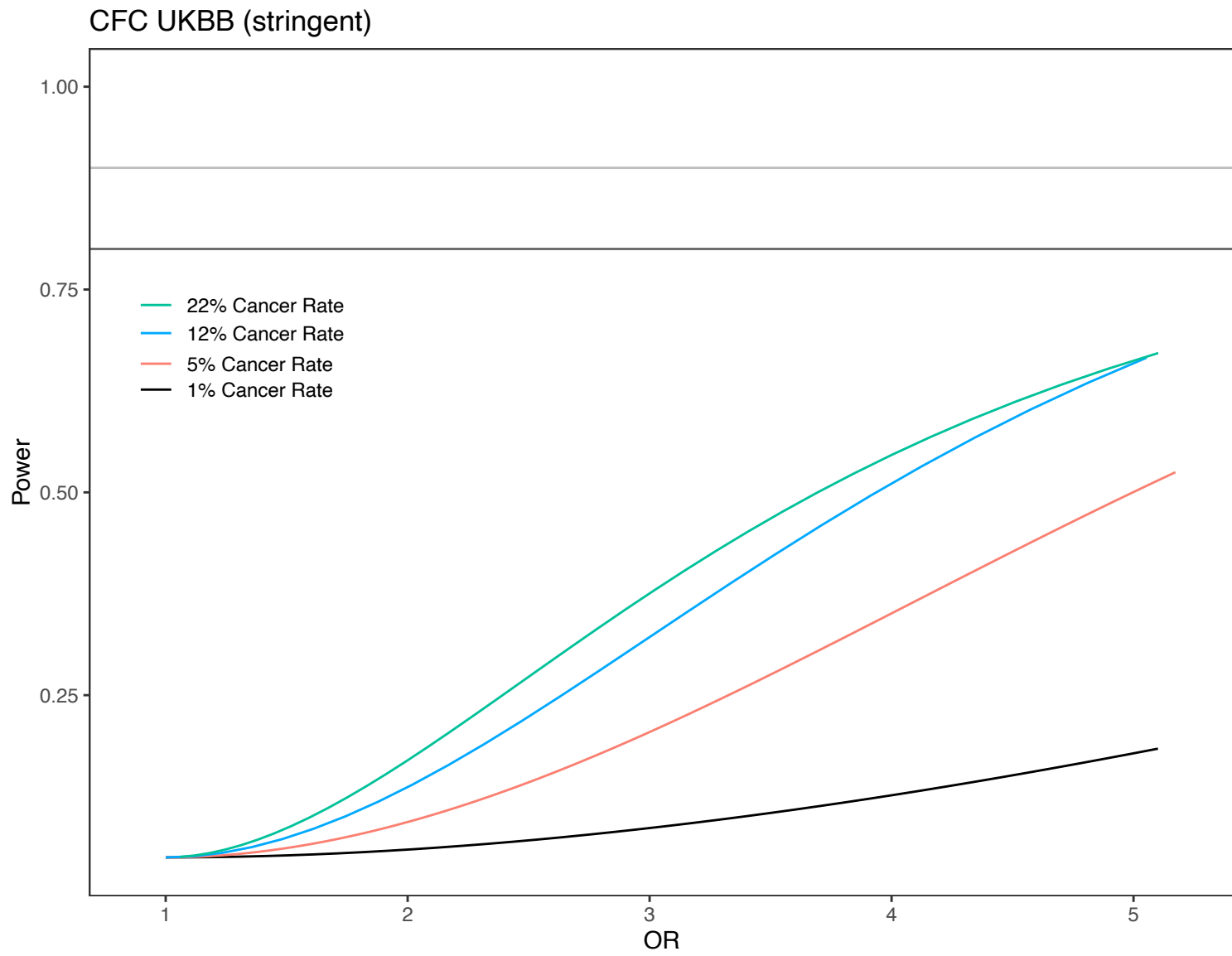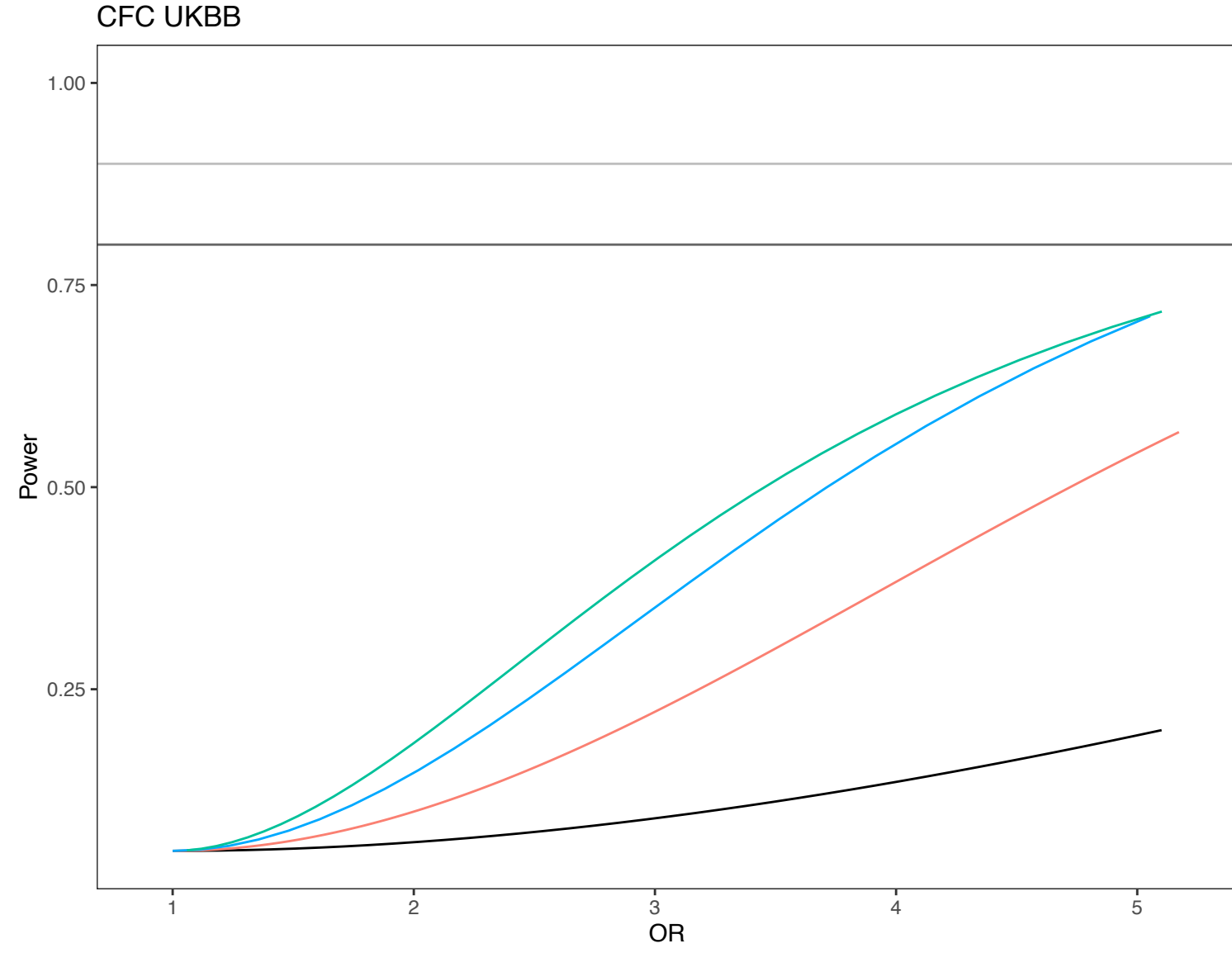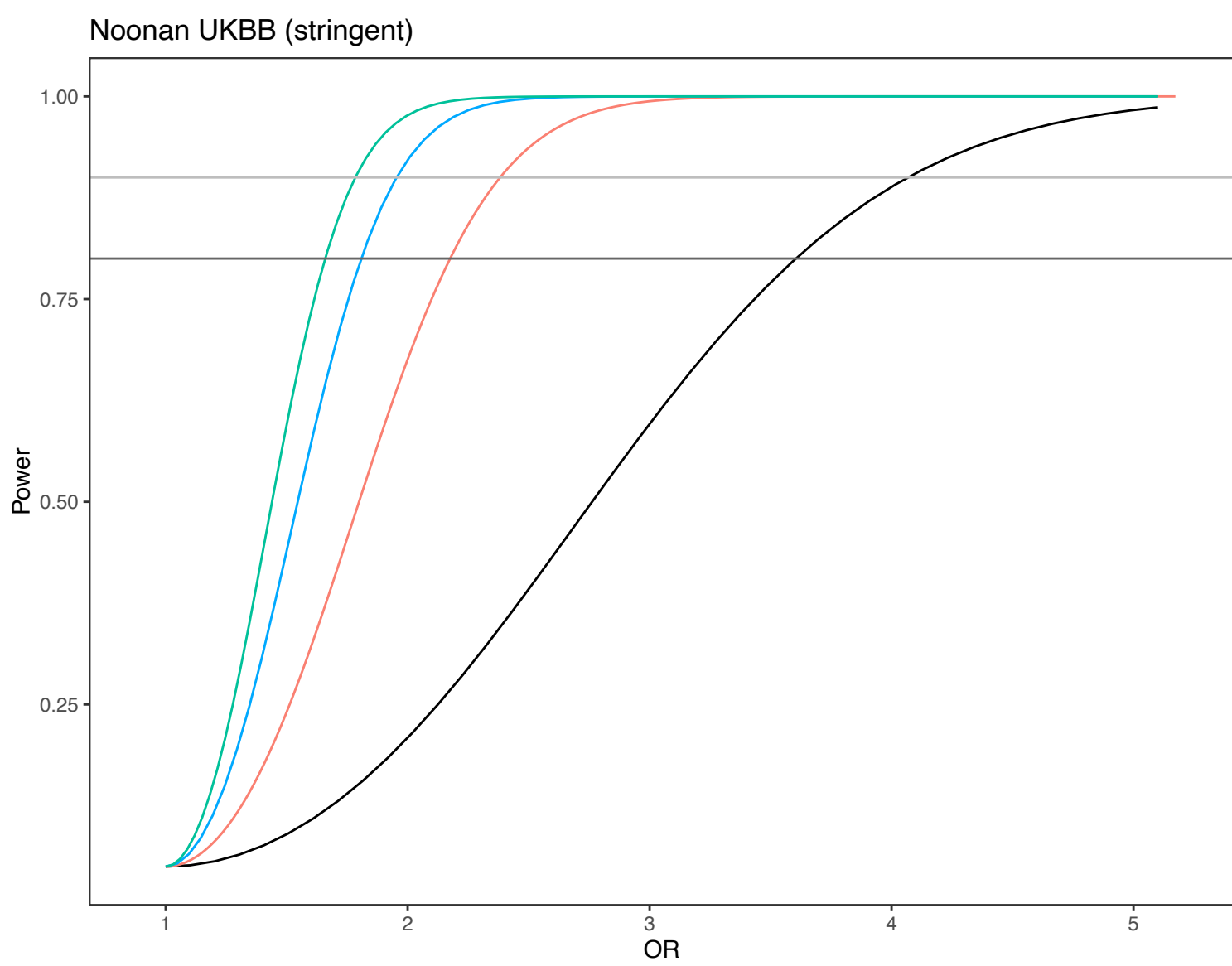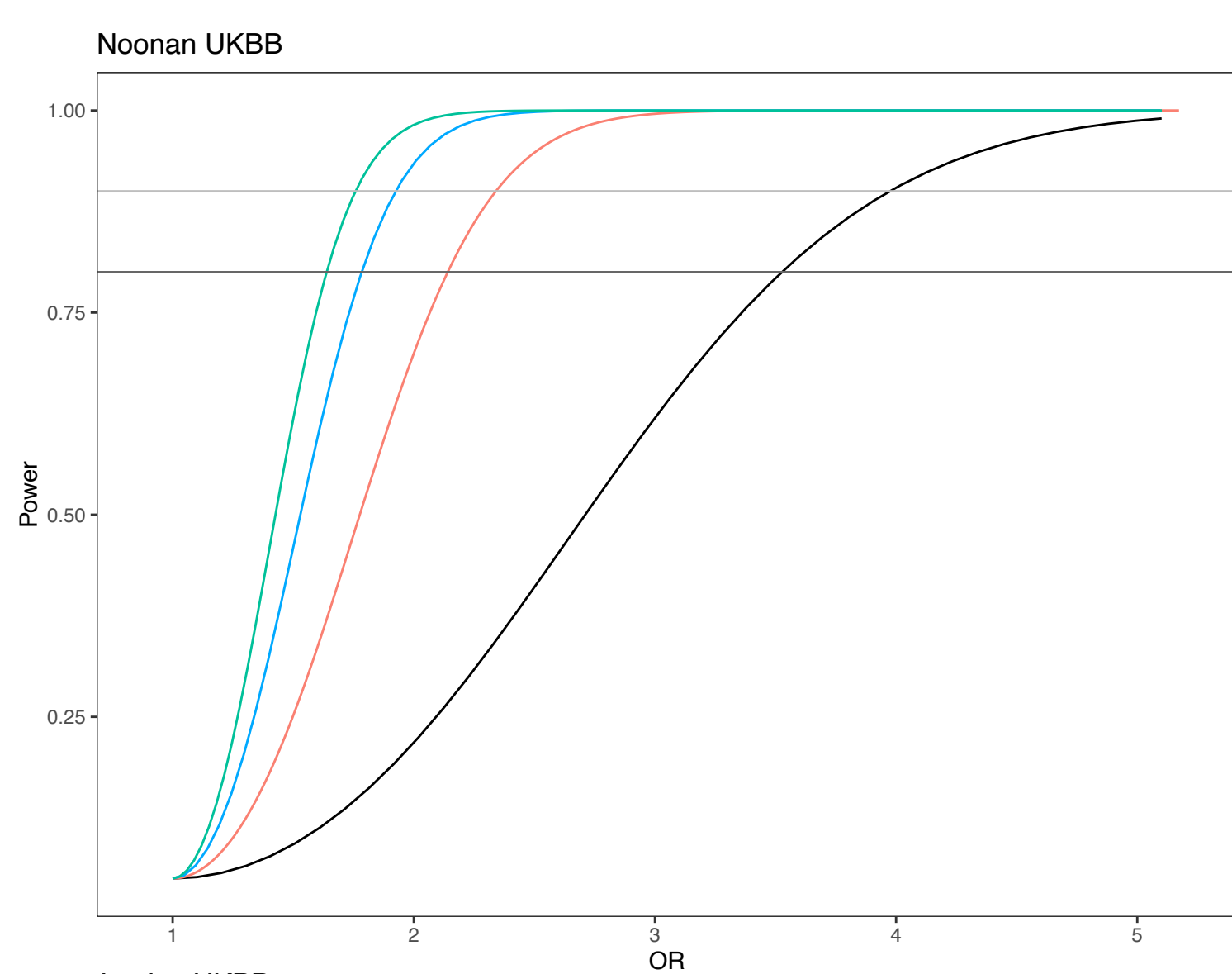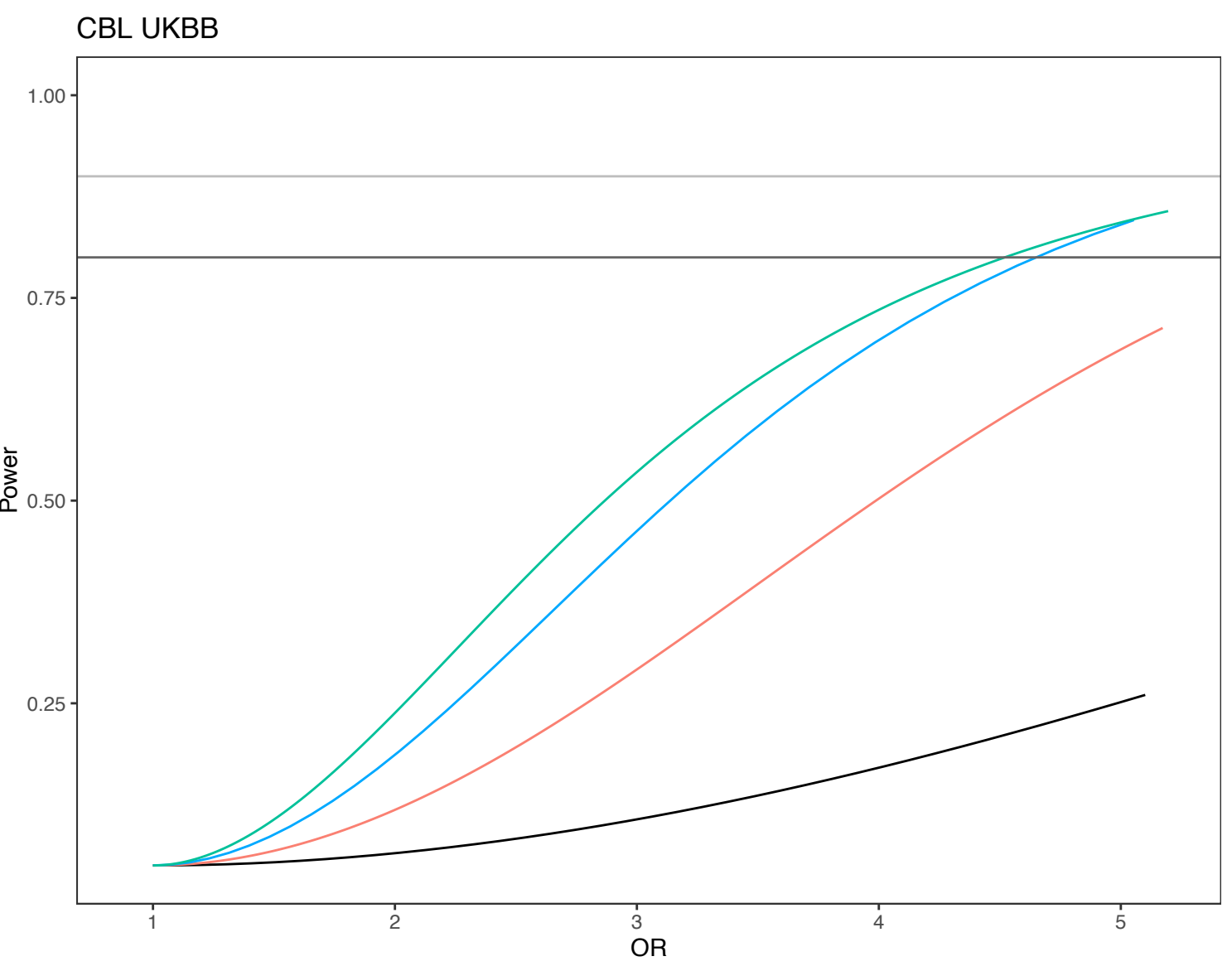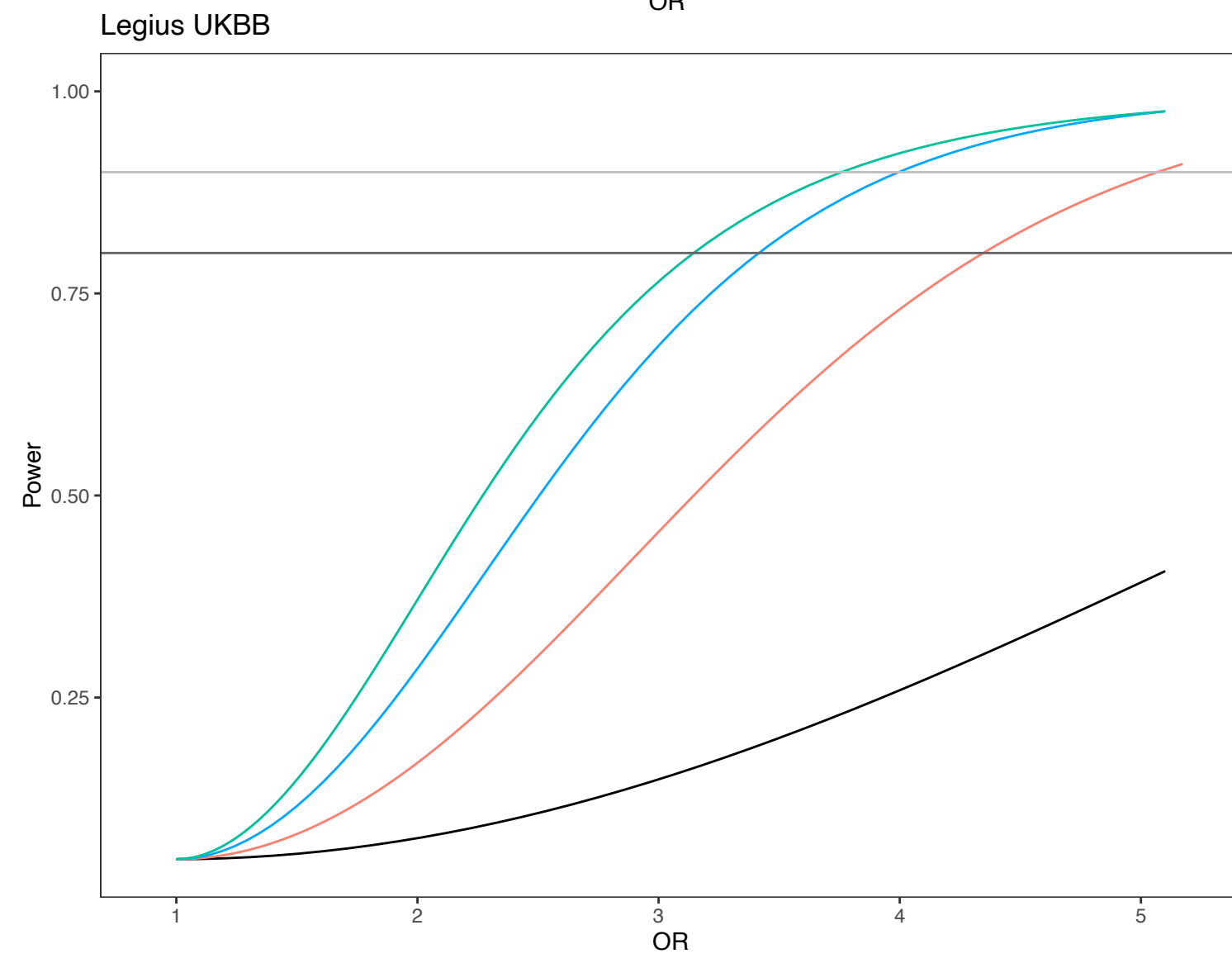

Supplement: Supplement 4 — Supplementary Figure 3. Power as a function of risk (odds ratio: OR) in UK Biobank for a range of cancer rates. Prevalence data from cohort-specific RASopathies (Table 1). Dark gray line represents 80% power, and light gray line represents 90% power. [file media-4.pdf]

Noonan BioMe

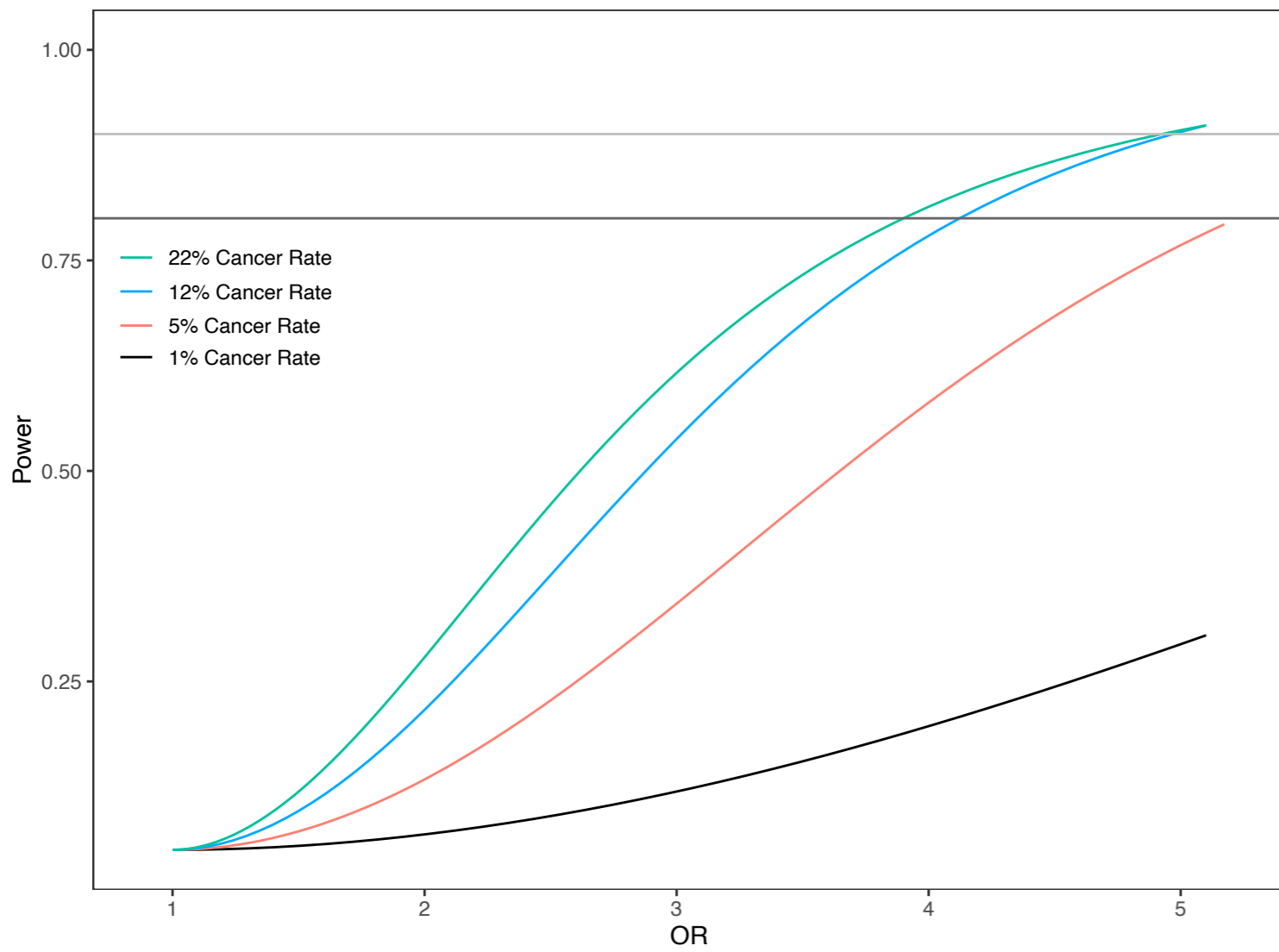

Noonan BioMe (stringent)

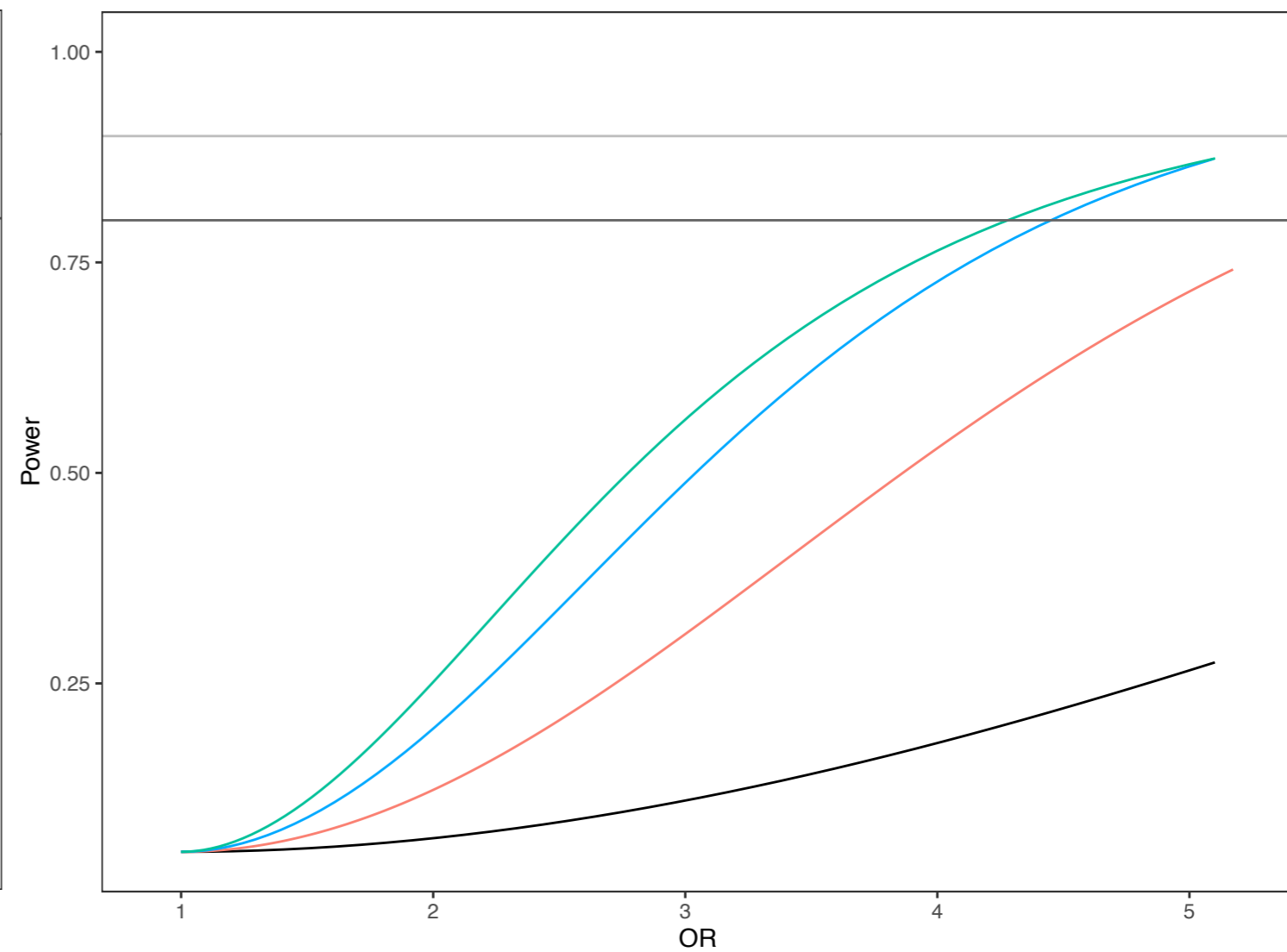

Supplement: Supplement 6 — Supplementary Figure 5. Power as a function of risk (odds ratio: OR) in BioMe for a range of cancer rates. Prevalence data from cohort-specific RASopathies (Table 1). Dark gray line represents 80% power, and light gray line represents 90% power. [file media-6.pdf]

A.

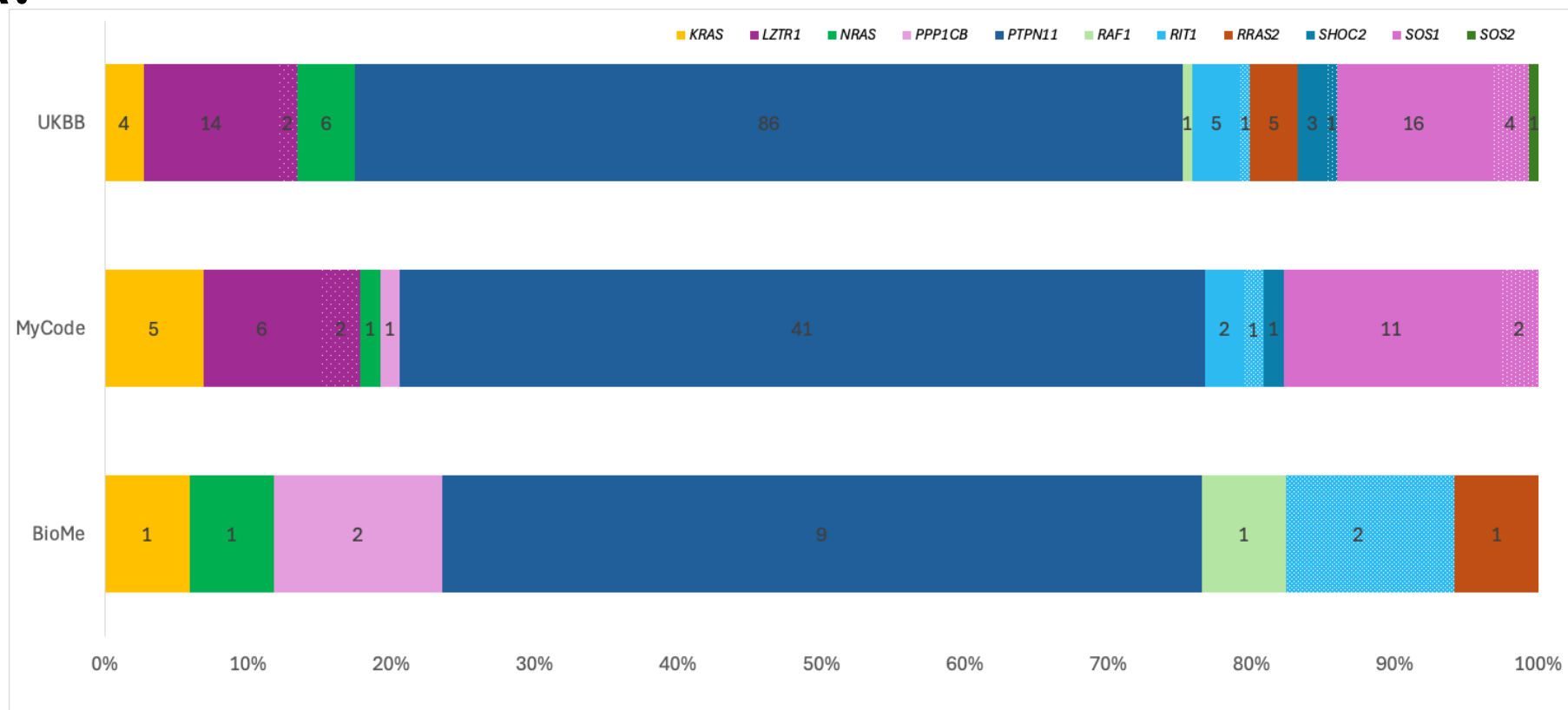

B.

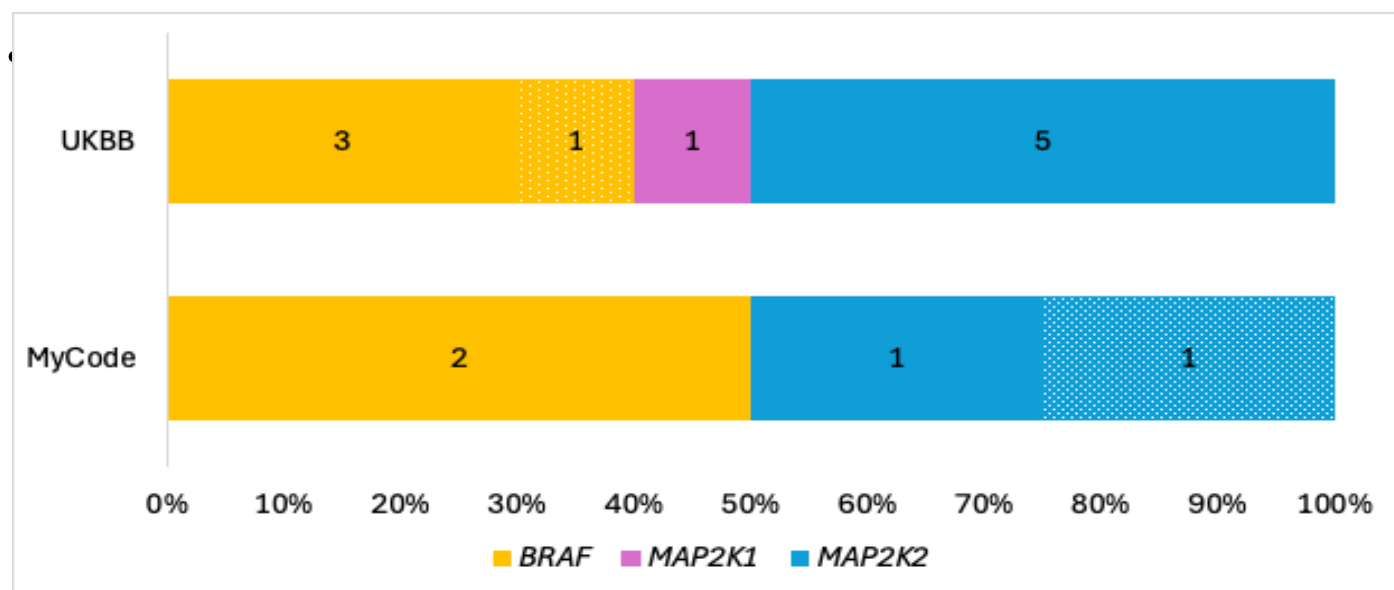

Supplement: Supplement 7 — Supplementary Figure 6. Absolute counts and percentages of P/LP variants observed in Noonan syndrome genes (panel A) and cardiofaciocutaneous syndrome genes (panel B). [file media-7.pdf]

A.

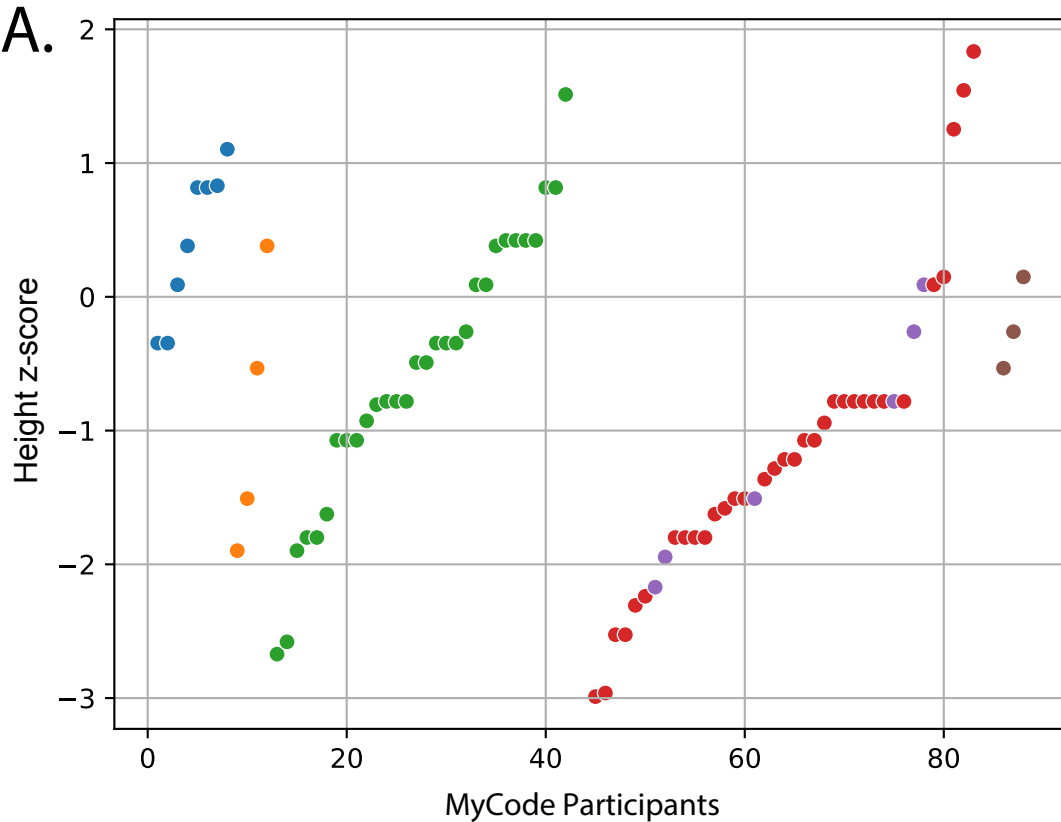

B.

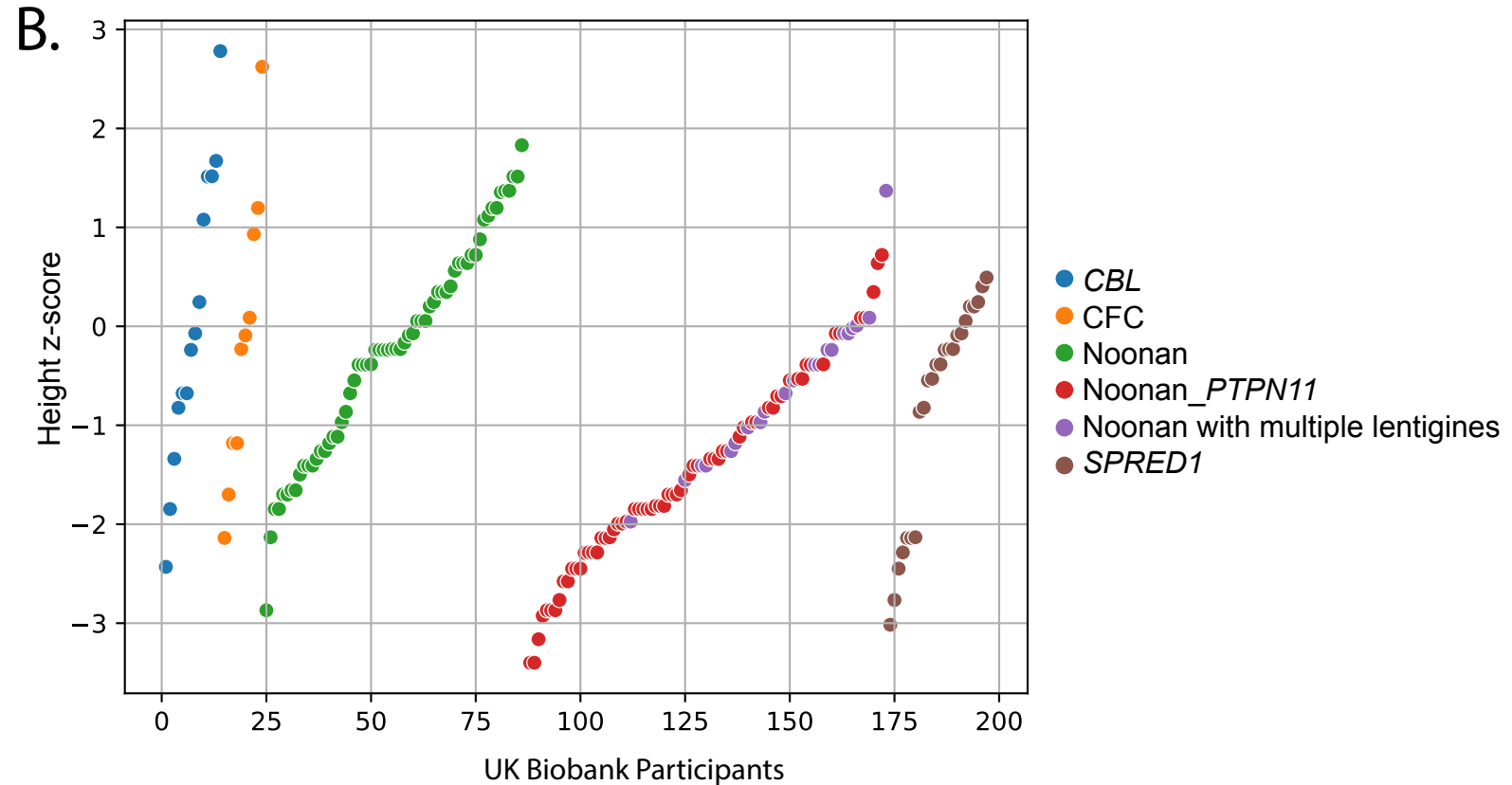

Supplement: Supplement 8 — Supplementary Figure 7. Height z-score in RASopathy heterozygotes in Geisinger (panel A) and UK Biobank (panel B). X-axis is a running sum of the number of participants for each heterozygote type. [file media-8.pdf]

# UK Biobank Cause of Death

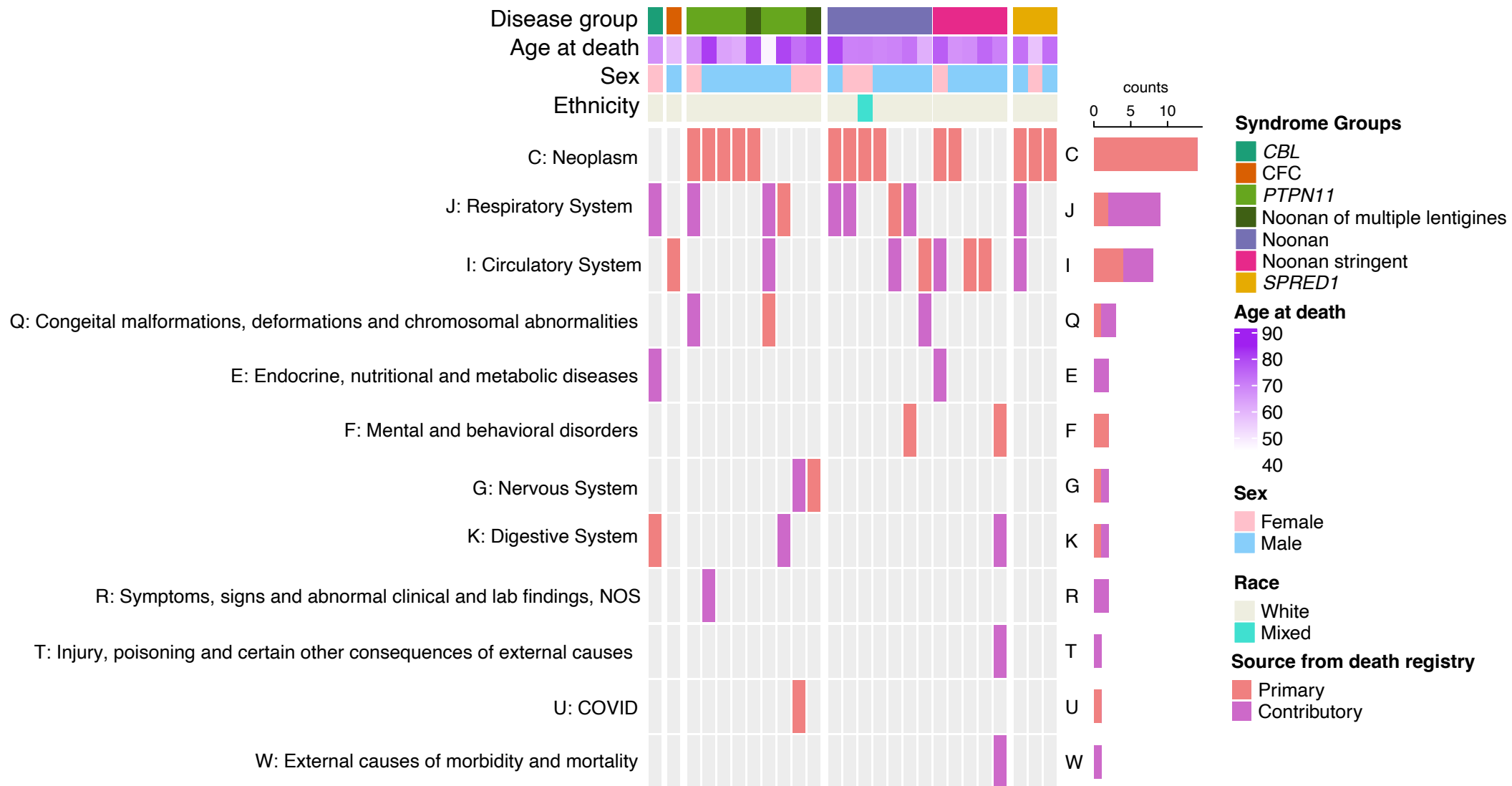

Supplement: Supplement 10 — Supplementary Figure 9. Top causes of death in RASopathy heterozygotes in UKBB. [file media-10.pdf]
