# Supplementary material for: Genomic ascertainment to quantify prevalence and cancer risk in adults with pathogenic and likely pathogenic germline variants in RASopathy genes
Source: medRxiv. 2024 Oct 11:2024.10.09.24314324. Preprint. [Version 1] doi: 10.1101/2024.10.09.24314324 (PMC11722494; doi:10.1101/2024.10.09.24314324)
Supplement: Supplement 5 — Supplementary Figure 4. Power as a function of risk (odds ratio: OR) in MyCode for a range of cancer rates. Prevalence data from cohort-specific RASopathies (Table 1). Dark gray line represents 80% power, and light gray line represents 90% power. [file media-5.pdf]

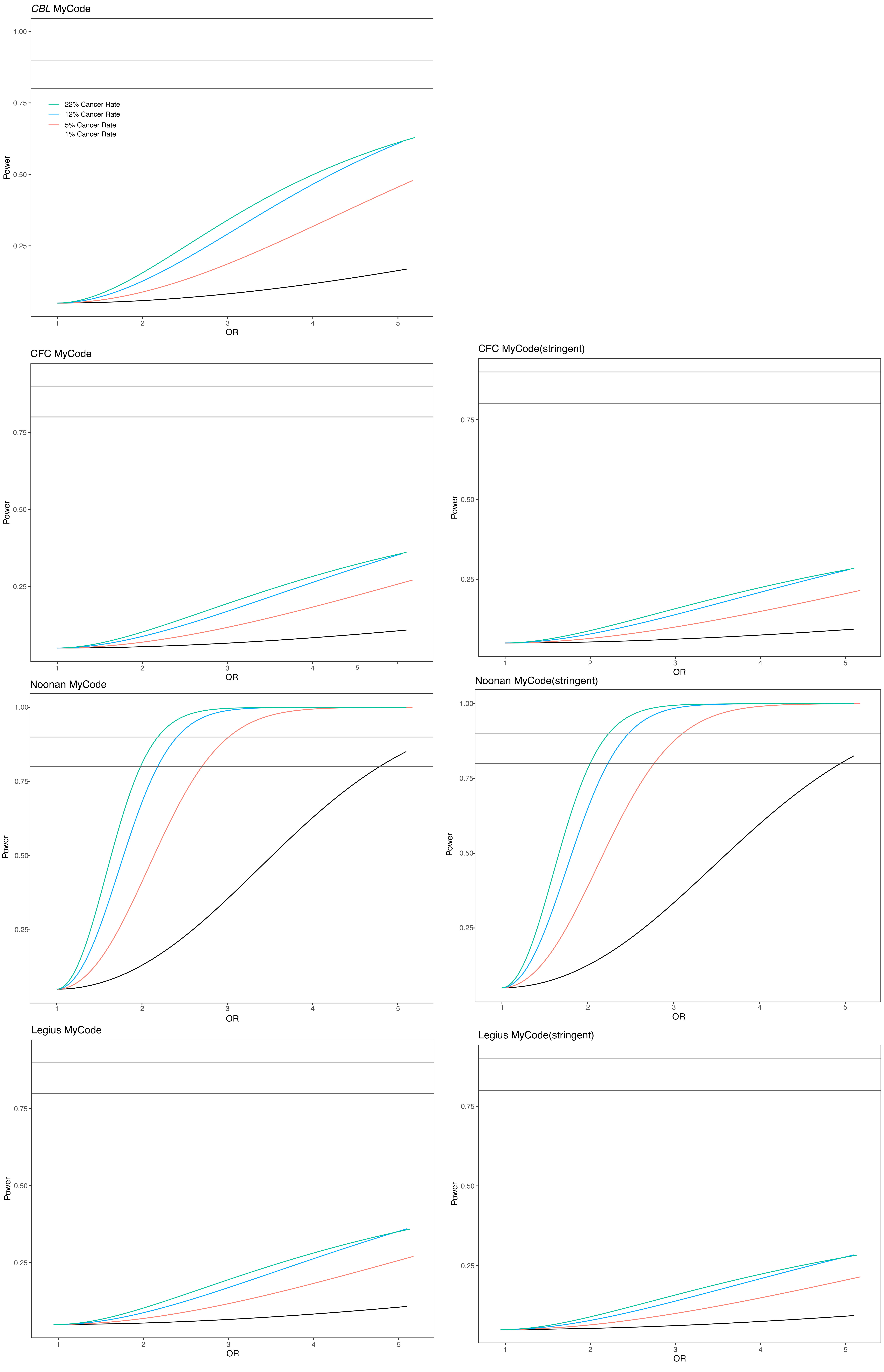
CBL MyCode
22% Cancer Rate
12% Cancer Rate
5% Cancer Rate
1% Cancer Rate
Power
OR
1.00
0.75
0.50
0.25
1
2
3
4
5
CFC MyCode
CFC MyCode(stringent)
Noonan MyCode
Noonan MyCode(stringent)
Legius MyCode
Legius MyCode(stringent)
