# Supplementary material for: Genomic ascertainment to quantify prevalence and cancer risk in adults with pathogenic and likely pathogenic germline variants in RASopathy genes
Source: medRxiv. 2024 Oct 11:2024.10.09.24314324. Preprint. [Version 1] doi: 10.1101/2024.10.09.24314324 (PMC11722494; doi:10.1101/2024.10.09.24314324)
Supplement: Supplement 9 — Supplementary Figure 8; Panels A-P. Gene-specific maps of pathogenic/likely pathogenic variants observed in participants with cancer and without cancer in all three biobanks. Variants in red in panel I (PTPN11) are known to be associated with Noonan syndrome and multiple lentigines. [file media-9.pdf]

Supplementary Figure 8: Lollipop plots of A)*BRAF* B)*CBL* C)*KRAS* D)*LZTR1* E)*MAP2K1* F)*MAP2K2* G)*NRAS* H)*PPP1CB* I)*PTPN11* J) *RAF1* K)*RIT1* L)*RRAS2* M)*SHOC2* N)*SOS1* O)*SOS2* P)*SPRED1*

A.

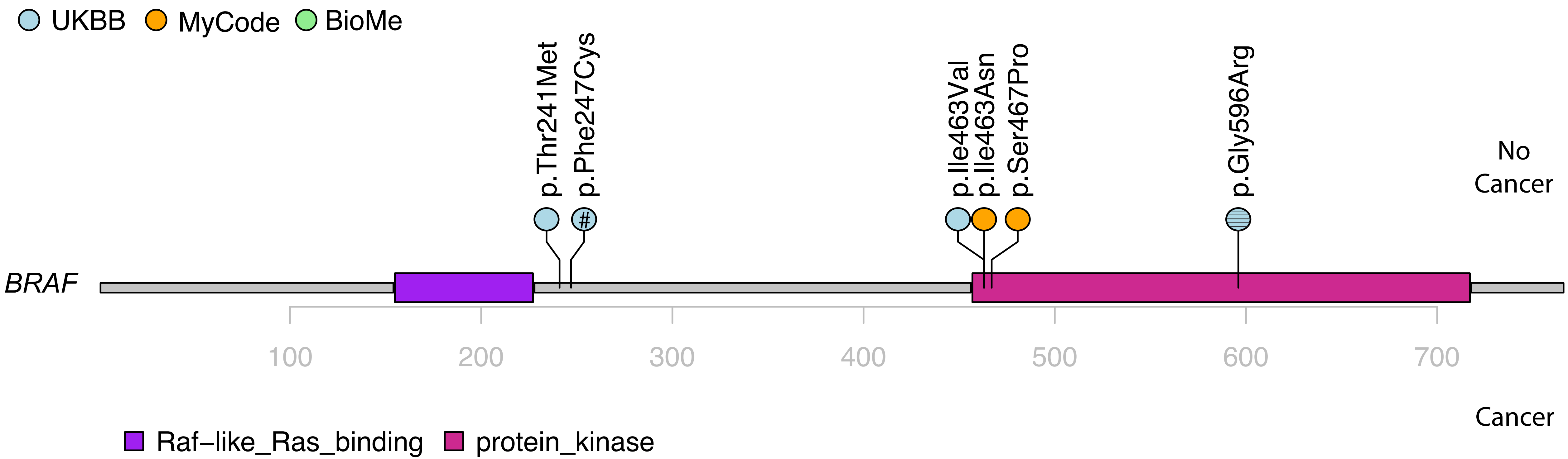

lines denotes variant that is dropped for stringent filter
# denotes death

B.

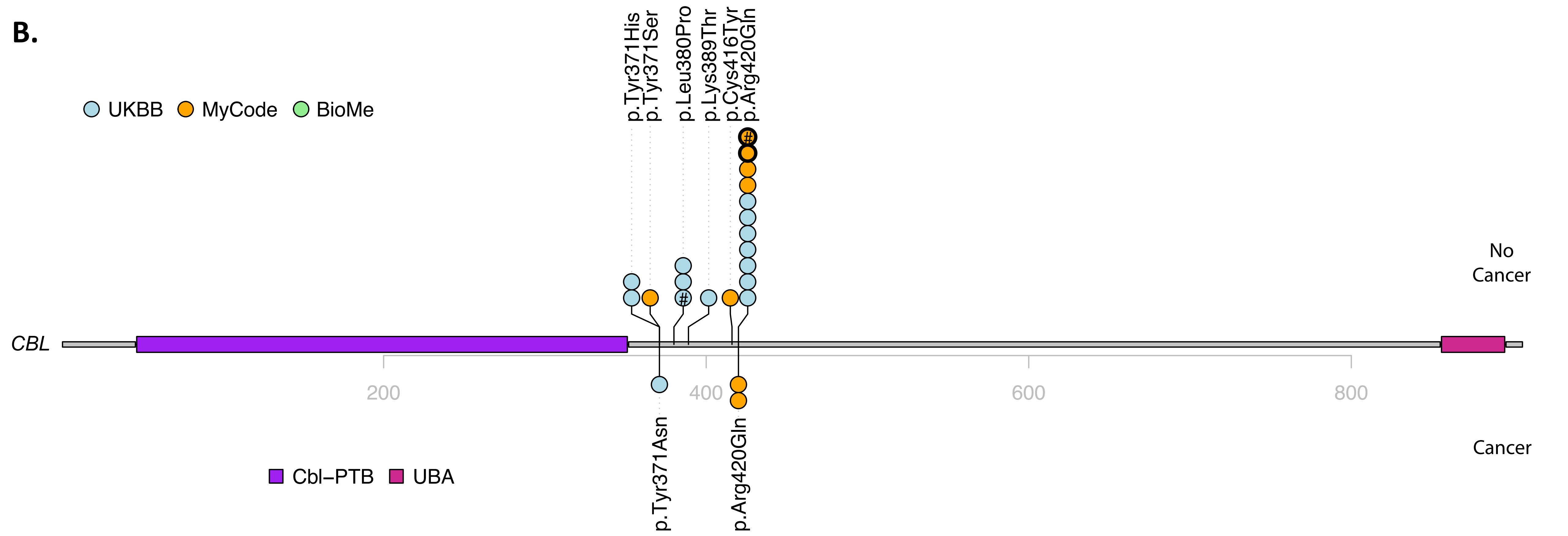

	HGVS p.	Cancer	Histology	Age at diagnosis	Database
1	p.Tyr371Asn	C44.9 Malignant neoplasm of skin	Basal cell carcinoma, NOS	78.4	UKBB
2	p.Arg420Gln	C49.6 Malignant neoplasm of connective and soft tissue of head, face and neck	Dermatofibrosarcoma protuberans, fibrosarcomatous	45	MyCode
3	p.Arg420Gln	C61.9 Malignant neoplasm of prostate	Adenocarcinoma, NOS	63	MyCode
3	p.Arg420Gln	C67.9 Malignant neoplasm of trigone of bladder	Papillary transitional cell carcinoma, non-invasive	77	MyCode

# denotes death
Bold outline represent related individuals

C.

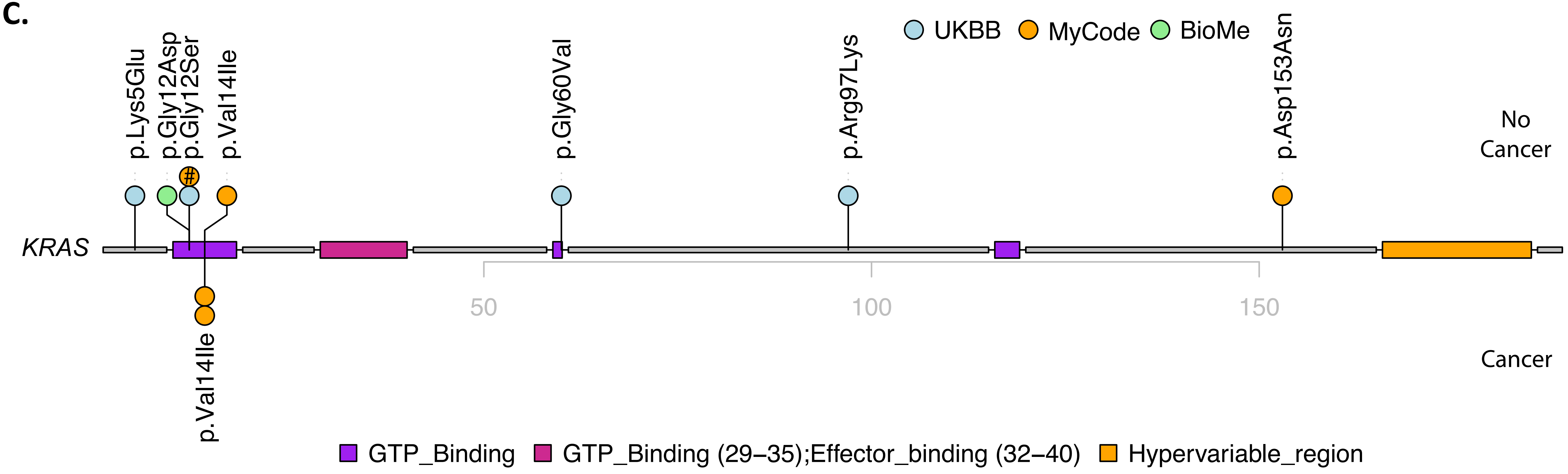

	HGVS p.	Cancer	Histology	Age at diagnosis	Database
1	p.Val14Ile	C44.3 Other specified malignant neoplasm of skin of unspecified lower limb, including hip	.	37.3	MyCode
1		C43.7 Malignant melanoma of unspecified lower limb, including hip	.	46	MyCode
2	p.Val14Ile	C44.3 Basal cell carcinoma of skin of nose	.	77.9	MyCode

D.

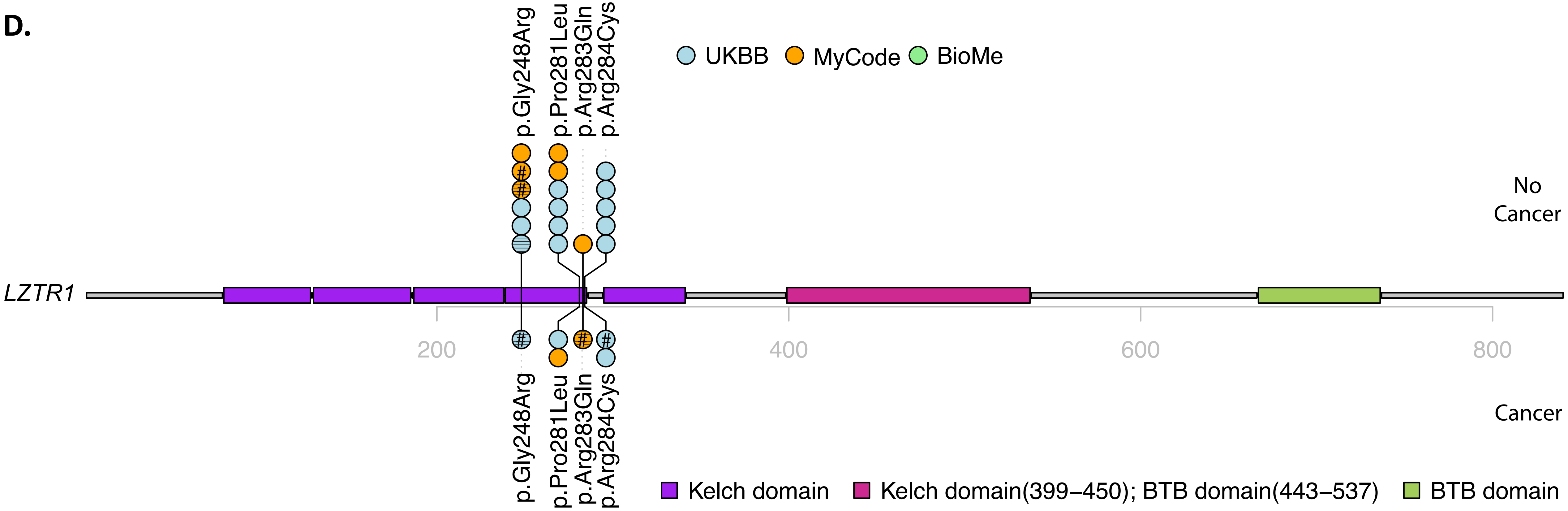

	HGVS p.	Cancer	Histology	Age at diagnosis	Database
1	p.Gly248Arg	C61 Malignant neoplasm of prostate	Adenocarcinoma, NOS	56.8	UKBB
2	p.Pro281Leu	C62.9 Testis, unspecified	Seminoma, NOS	50.2	UKBB
3	p.Pro281Leu	C75.1 Malignant neoplasm of pineal gland	adenoma, NOS	74	MyCode
3		C72.5Malignant neoplasm of unspecified cranial nerve	.	76	MyCode
4	p.Arg283Gln	C61.9 Malignant neoplasm of prostate	Adenocarcinoma, NOS	67	MyCode
4		C44 Basal cell carcinoma	.	85	MyCode
5	p.Arg284Cys	C44.9 Malignant neoplasm of skin, unspecified; C44.2 Skin of ear and external auricular canal	Basal cell carcinoma, NOS	70.7	UKBB
6	p.Arg284Cys	C67.9 Bladder, unspecified	Transitional cell carcinoma, NOS	58.1	UKBB
6		C44.4 Skin of scalp and neck	Basal cell carcinoma, NOS	80.5	UKBB
6		C34.9 Bronchus or lung, unspecified; C34.1 Upper lobe, bronchus or lung	.	81.6	UKBB
6		C79.9 Secondary malignant neoplasm, unspecified site	.	81	UKBB

E.

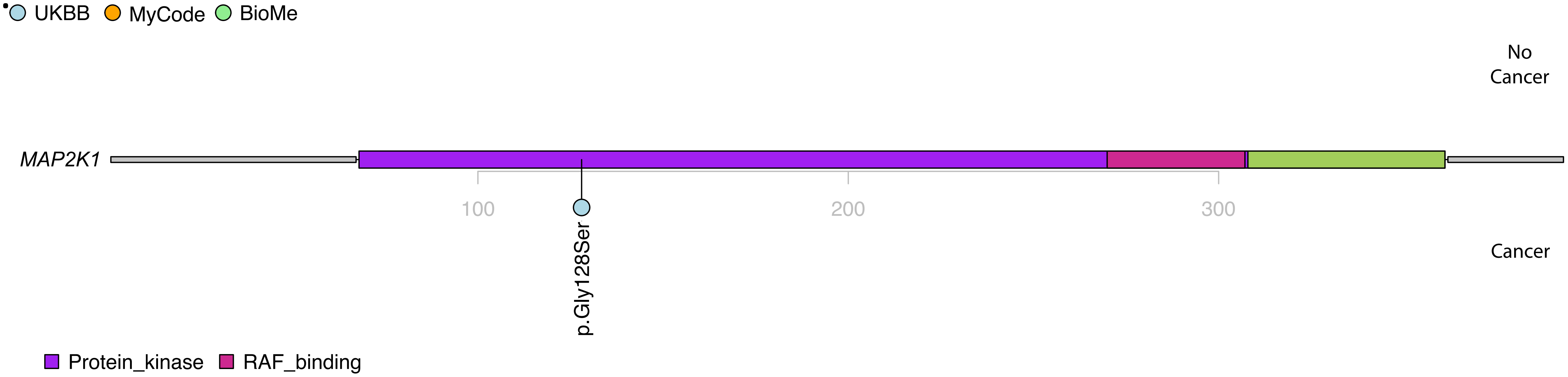

	HGVS p.	Cancer	Histology	Age at diagnosis	Database
1	p.Gly128Ser	C50.9 Breast, unspecified; C50.3 Lower-inner quadrant of breast	Infiltrating duct carcinoma, NOS	65.8	UKBB
		C77.3 Axillary and upper limb lymph nodes	.	66	UKBB

F.

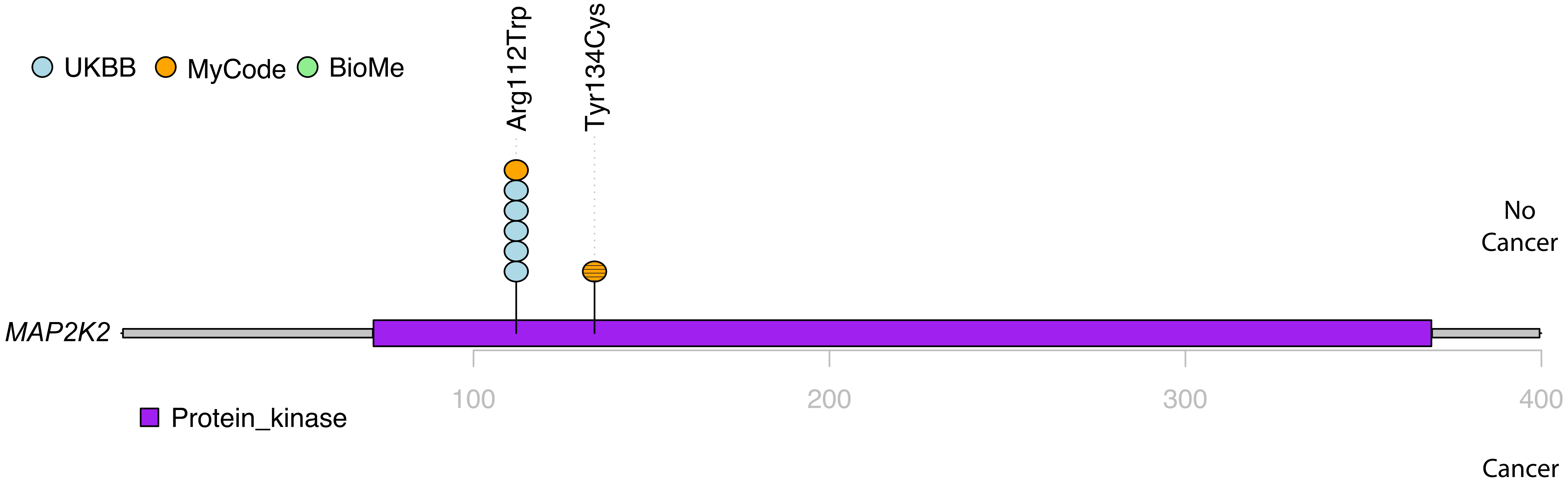
UKBB
MyCode
BioMe
Arg112Trp
Tyr134Cys
No Cancer
MAP2K2
100
200
300
400
Protein_kinase
Cancer

G.

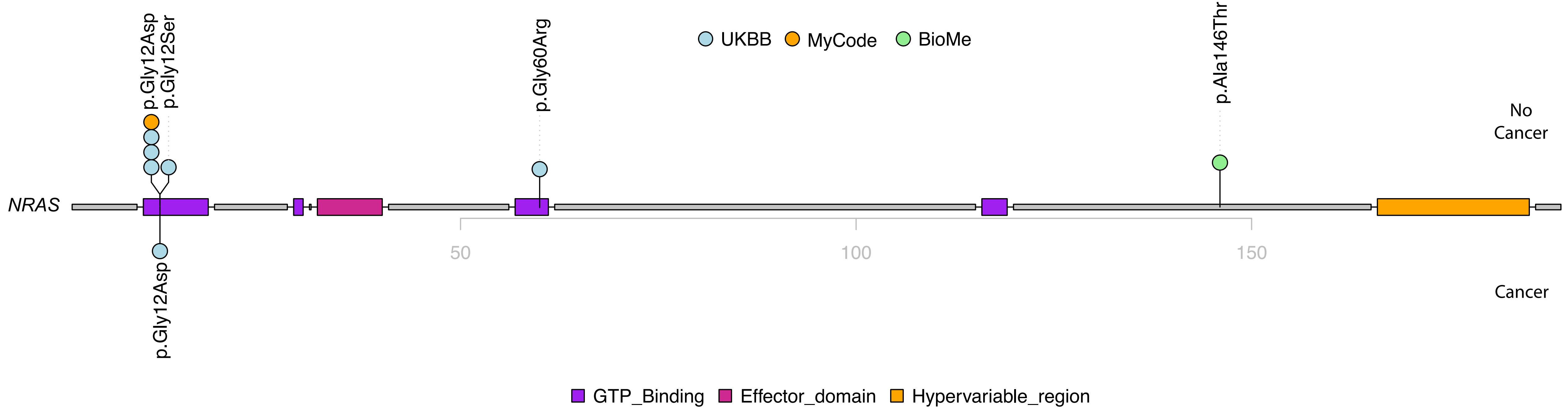

	HGVS p.	Cancer	Histology	Age at diagnosis	Database
1	p.Gly12Asp	C44.3 Skin of other and unspecified parts of face	Basal cell carcinoma, NOS	49.4	UKBB

H.

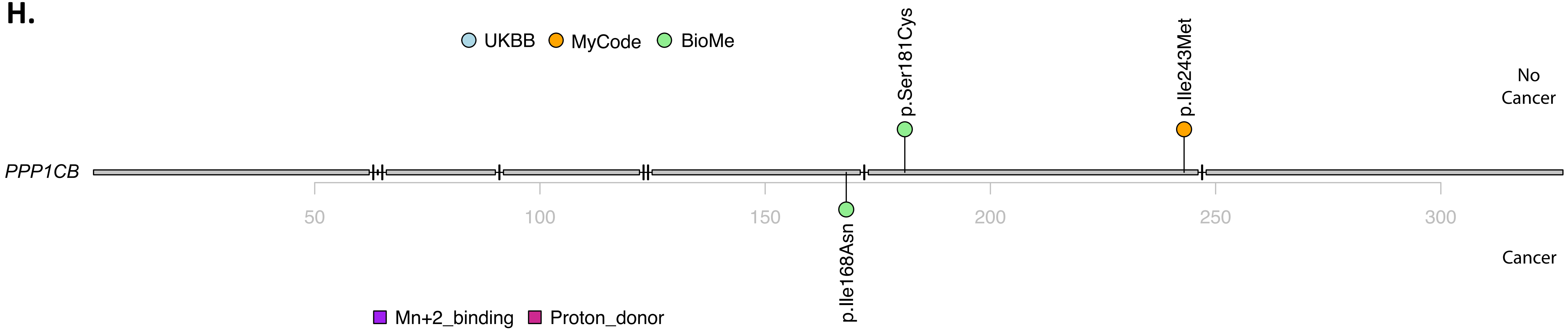

	HGVS p.	Cancer	Histology	Age at diagnosis	Database
1	p.Ile168Asn	C50.9 Malignant neoplasm of unspecified site of unspecified female breast	.	50	BioMe
1		C34.9 Malignant neoplasm of unspecified part of unspecified bronchus or lung	.	55	BioMe

I.

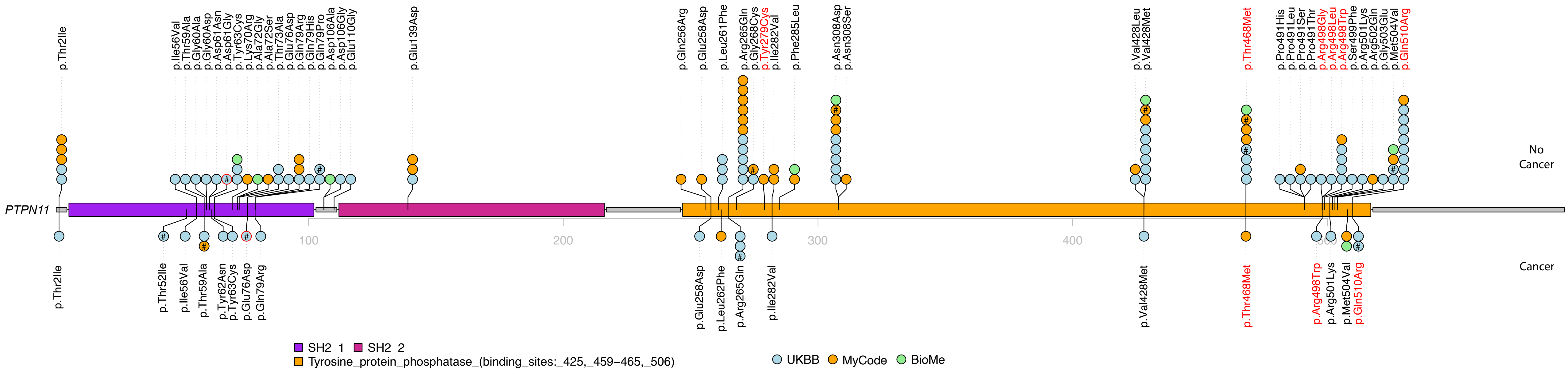
p.Thr2Ile
p.Ile56Val
p.Thr59Ala
p.Gly60Ala
p.Gly60Asp
p.Asp61Asn
p.Asp61Gly
p.Tyr63Cys
p.Lys70Arg
p.Ala72Gly
p.Ala72Ser
p.Thr73Ala
p.Glu76Asp
p.Gln79Arg
p.Gln79His
p.Gln79Pro
p.Asp106Ala
p.Asp106Gly
p.Glu110Gly
p.Glu139Asp
p.Gln256Arg
p.Glu258Asp
p.Leu261Phe
p.Arg265Gln
p.Gly268Cys
p.Tyr279Cys
p.Ile282Val
p.Phe285Leu
p.Asn308Asp
p.Asn308Ser
p.Val428Leu
p.Val428Met
p.Thr468Met
p.Pro491His
p.Pro491Leu
p.Pro491Ser
p.Pro491Thr
p.Arg498Gly
p.Arg498Leu
p.Arg498Trp
p.Ser499Phe
p.Arg501Lys
p.Arg502Gln
p.Gly503Glu
p.Met504Val
p.Gln510Arg
No Cancer
PTPN11
100
200
300
400
500
Cancer
p.Thr2Ile
p.Thr52Ile
p.Ile56Val
p.Thr59Ala
p.Tyr62Asn
p.Tyr63Cys
p.Glu76Asp
p.Gln79Arg
p.Glu258Asp
p.Leu262Phe
p.Arg265Gln
p.Ile282Val
p.Val428Met
p.Thr468Met
p.Arg498Trp
p.Arg501Lys
p.Met504Val
p.Gln510Arg
SH2_1
SH2_2
Tyrosine_protein_phosphatase_(binding_sites:_425,_459–465,_506)
UKBB
MyCode
BioMe

	HGVS p.	Cancer	Histology	Age at diagnosis	Database
1	p.Thr2Ile	C73 Malignant neoplasm of thyroid gland	Papillary adenocarcinoma, NOS	59.1	UKBB
2	p.Thr52Ile	C18.0 Caecum	Neoplasm, malignant	80.1	UKBB
3	p.Ile56Val	C16.2 Body of stomach	.	63.4	UKBB
3		C79.5 Secondry malignant neoplasm of bone and bone marrow	.	64	UKBB
4	p.Thr59Ala	C61 Malignant neoplasm of prostate	Adenocarcinoma, NOS	63.4	UKBB
5	p.Thr59Ala	C34.1 Malignant neoplasm of unspecified part of unspecified bronchus or lung	Squamous cell carcinoma, NOS	83	MyCode
5		C61.9 Malignant neoplasm of prostate	Adenocarcinoma, NOS	74	MyCode
6	p.Tyr62Asn	C20 Malignant neoplasm of rectum	Adenocarcinoma, NOS	62.2	UKBB
7	p.Tyr63Cys	C44.5 Skin of trunk	Basal cell carcinoma, NOS	43.8	UKBB
7		C43.5 Malignant melanoma of trunk	.	55	UKBB
7		C44.4 Skin of scalp and neck	.	59	UKBB
7		C44.5 Skin of trunk, C44.6 Skin of upper limb, including shoulder	.	54	UKBB
7		C97 Malignant neoplasms of independent (primary) multiple sites	.	66	UKBB
8	p.Glu76Asp	C71.2 Temporal lobe	Glioblastoma, NOS	63.4	UKBB
9	p.Gln79Arg	C50.9 Breast	.	62	UKBB
9		1869 Malignant neoplasm of testis, other and unspecified	.	27.8	UKBB
10	p.Glu258Asp	C20 Malignant neoplasm of rectum	Adenocarcinoma, NOS	69.9	UKBB
11	p.Leu262Phe	C20 Malignant neoplasm of rectum	.	61.1	MyCode
12	p.Arg265Gln	C19 Malignant neoplasm of rectosigmoid junction	Adenocarcinoma, NOS	59.6	UKBB
12		C20 Malignant neoplasm of rectum		60	UKBB
12		C78.0 Secondary malignant neoplasm of lung; C78.7 Secondary malignant neoplasm of liver		60	UKBB
13	p.Arg265Gln	C34.1 Upper lobe, bronchus or lung	Adenocarcinoma, NOS	46.5	UKBB
13		C77.1 Intrathoracic lymph nodes		47	UKBB
14	p.Arg265Gln	C61 Malignant neoplasm of prostate	Adenocarcinoma, NOS	68.6	UKBB
15	p.Ile282Val	C44.9 Malignant neoplasm of skin, unspecified	Basal cell carcinoma, NOS	64.7	UKBB
16	p.Val428Met	C74.1 Medulla of adrenal gland	Neoplasm, malignant	41.2	UKBB
17	p.Thr468Met	C44.5 Skin of trunk	Malignant melanoma, NOS	41	MyCode
17		C44.3 Skin of other and unspecified parts of face	Lentigo maligna	55	MyCode
17		C44.6 Skin of upper limb and shoulder; C44.7 Skin of lower limb and hip	Melanoma in situ	59	MyCode
17		C44.6 Skin of upper limb and shoulder	Lentigo maligna	44	MyCode
18	p.Arg498Trp	C43.6 Malignant melanoma of upper limb, including shoulder	.	69	UKBB
19	p.Arg501Lys	C62.9 Testis, unspecified	Seminoma, NOS	57.8	UKBB
20	p.Met508Val	C44.3 Basal cell carcinoma ok skin of nose, C44.9 Basal cell carcinoma of skin, unspecified	.	68	BioMe
21	p.Met508Val	C18.1 Malignant neoplasm of appendix	.	76.9	MyCode
22	p.Gln510Arg	C44.3 Skin of other and unspecified parts of face	Basal cell carcinoma, NOS	58.1	UKBB
22		C17.9 Small intestine, unspecified	Carcinoid tumour, malignant	74.2	UKBB
22		C44.6 Skin of upper limb, including shoulder	Squamous cell carcinoma, NOS	77.4	UKBB
22		C43.5 Malignant neoplasm of trunk	.	73	UKBB
22		C44.4 Skin of scale and neck, C44.6 Skin of upper limb, including shoulder	.	78	UKBB
22		C44.5 Skin of trunk	.	74	UKBB
22		C75.9 Endocrine gland, unspecified	.	78	UKBB
22		C78.6 Secondary malignant neoplasm of retroperitoneum and peritoneum, C78.7 Secondary malignant neoplasm of liver	.	75	UKBB
22		C97 Malignant neoplasms of independent (primary) multiple sites	.	78	UKBB
23	p.Gln510Arg	C43.4 Malignant melanoma of scalp and neck	Lentigo maligna melanoma	57.1	UKBB

J.

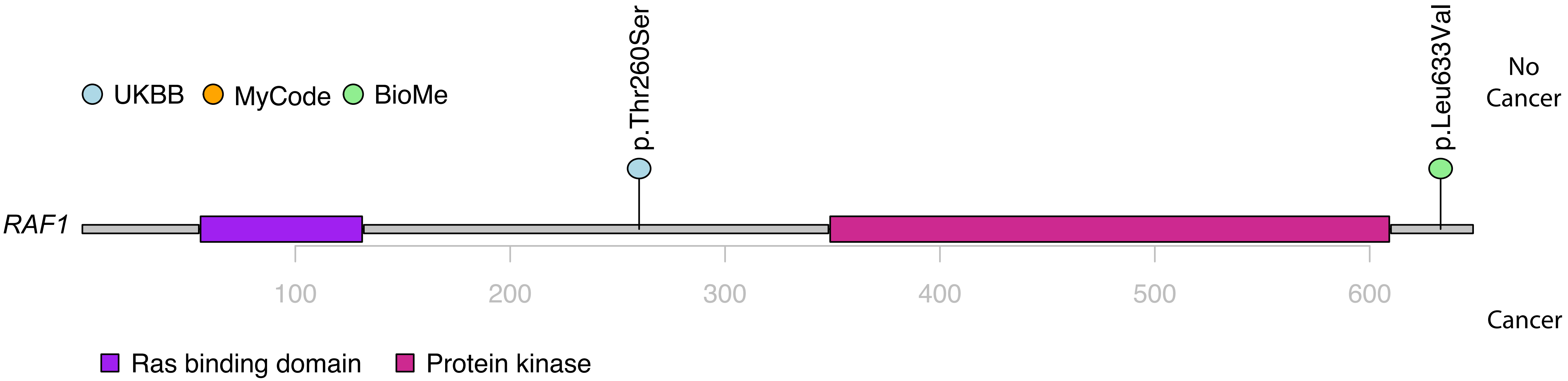

UKBB
MyCode
BioMe
p.Thr260Ser
p.Leu633Val
No Cancer
RAF1
100
200
300
400
500
600
Cancer
Ras binding domain
Protein kinase

K.

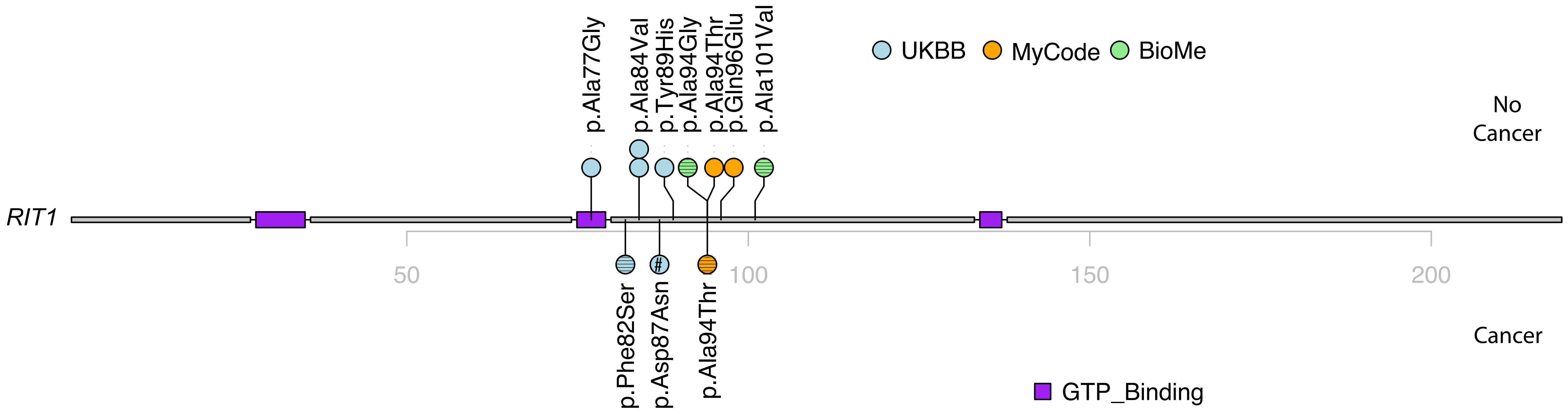

	HGVS p.	Cancer	Histology	Age at diagnosis	Database
1	p.Phe82Ser	C44.9 Malignant neoplasm of skin, unspecified	Squamous cell carcinoma, NOS	79.2	UKBB
1		C44.9 Malignant neoplasm of skin, unspecified	Basal cell carcinoma, NOS	80.5	UKBB
2	p.Asp87Asn	C34.1 Upper lobe, bronchus or lung; C34.9 Bronchus or lung, unspecified	Small cell carcinoma, NOS	69.2	UKBB
2		C78.7 Secondary malignant neoplasm of liver	.	69	UKBB
2		C79.3 Secondary malignant neoplasm of brain and cerebral meninges; C79.5 Secondary malignant neoplasm of bone and bone marrow	.	70	UKBB
3	p.Ala94Thr	C44.5 Unspecified malignant neoplasm of skin of other part of trunk	.	72.6	MyCode
3		C44.2 Basal cell carcinoma of skin of unspecified ear and external auricular canal	.	75.8	MyCode
3		C44.3 Squamous cell carcinoma of skin of other parts of face	.	80.2	MyCode

L.

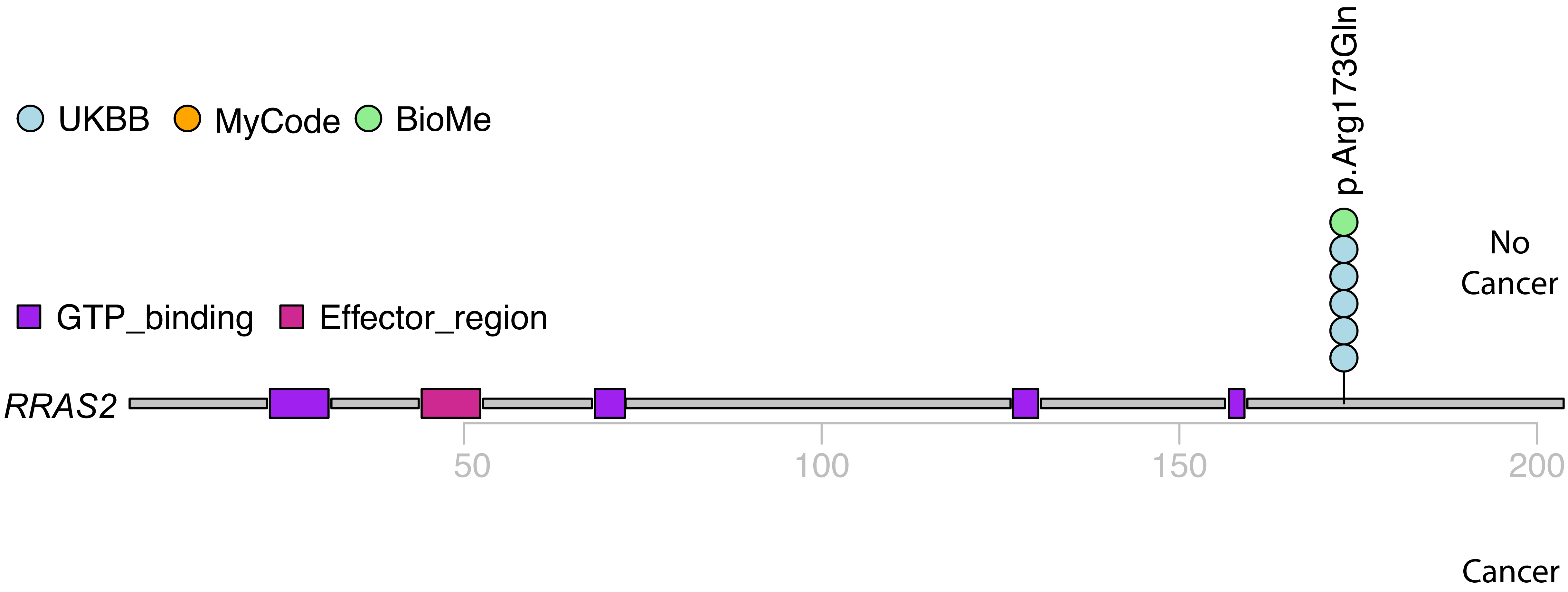

UKBB
MyCode
BioMe
GTP_binding
Effector_region
p.Arg173Gln
No Cancer
RRAS2
50
100
150
200
Cancer

M.

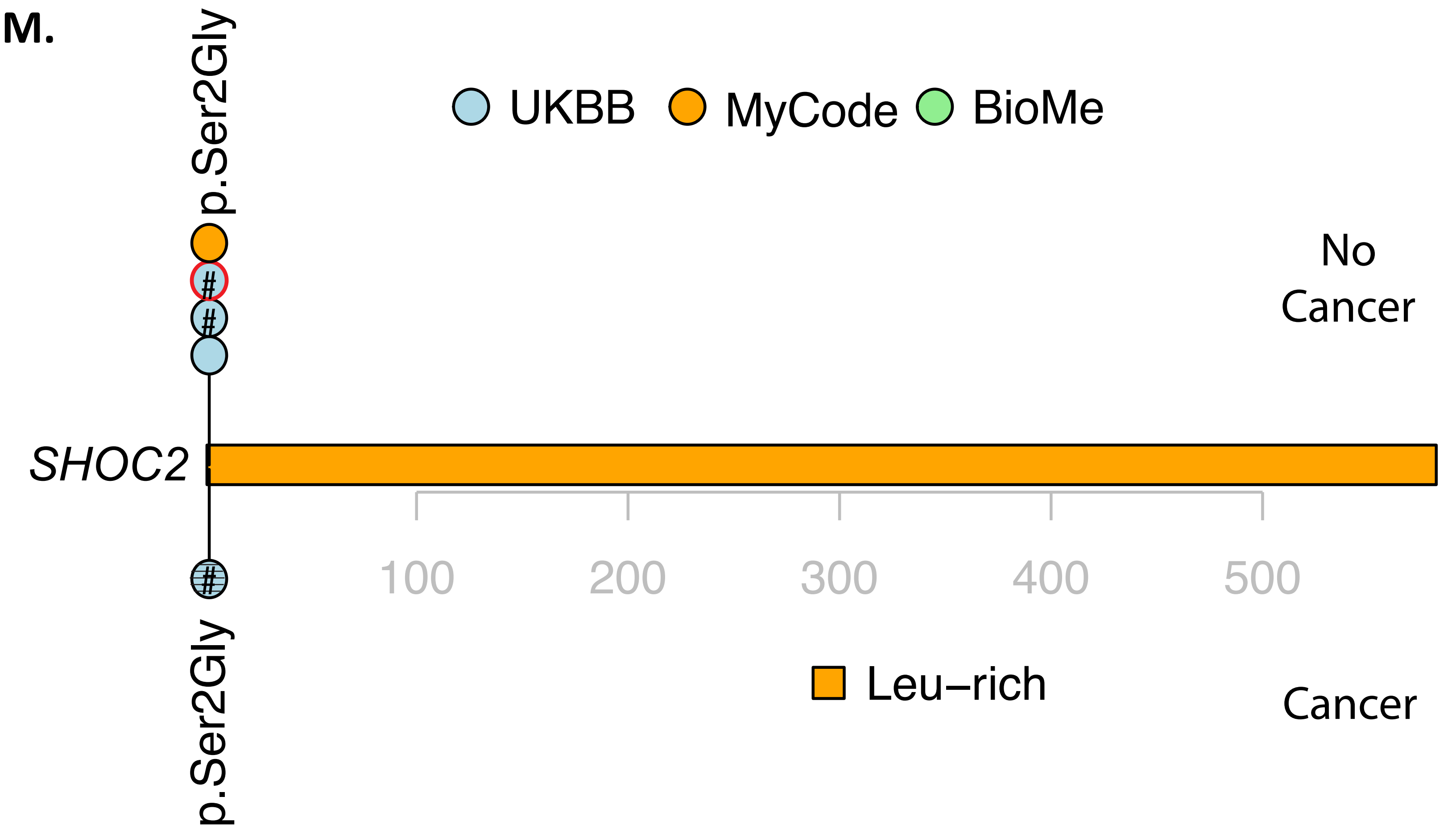

lines denotes variant that is dropped for stringent filter

# denotes death

Red outline represents individuals with Q87

	HGVS p.	Cancer	Histology	Age at diagnosis	Database
1	p.Ser2Gly	C34.2 Middle lobe, bronchus or lung; C34.9 Bronchus or lung, unspecified	Adenocarcinoma, NOS	76.1	UKBB
1		C79.5 Secondary malignant neoplasm of bone and bone marrow	.	77	UKBB

N.

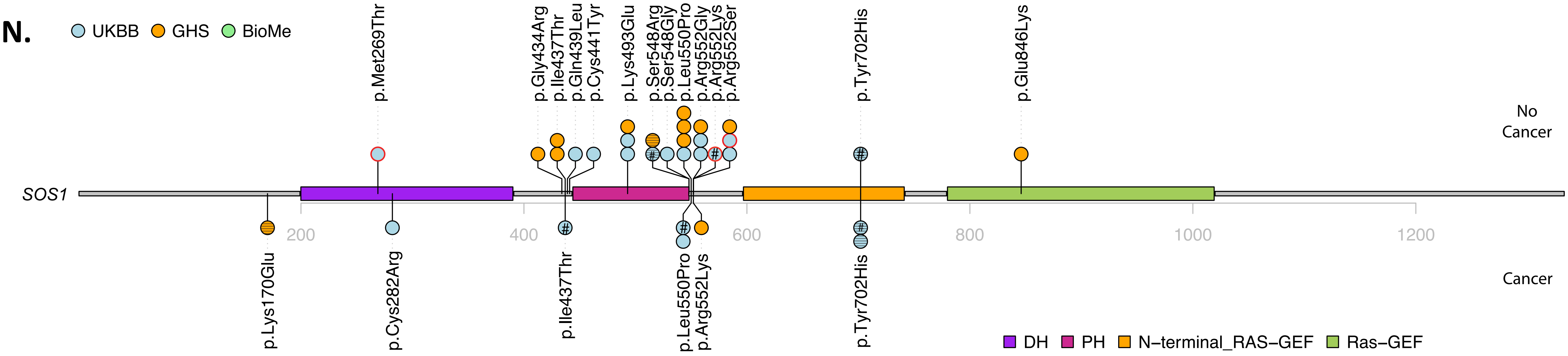

	HGVS p.	Cancer	Histology	Age at diagnosis	Database
1	p.Lys170Glu	C56.9 Malignant neoplasm of unspecified ovary	Low-grade serous carcinoma	67	MyCode
2	p.Cys282Arg	C50.4 Upper-outer quadrant of breast	Infiltrating duct carcinoma, NOS	50.8	UKBB
3	p.Ile437Thr	C34.1 Upper lobe, bronchus or lung; C34.9 Bronchus or lung, unspecified	Adenocarcinoma, NOS	68.7	UKBB
3		C79.3 Secondary malignant neoplasm of brain and cerebral meninges	.	68	UKBB
3		C79.5 Secondary malignant neoplasm of bone and bone marrow	.	69	UKBB
3		C80.9 Malignant neoplasm, unspecified	.	68	UKBB
4	p.Leu550Pro	C80 Malignant neoplasm without specification of site	.	66.8	UKBB
4		C56 Malignant neoplasm of ovary	.	67	UKBB
4		C78.6 Secondary malignant neoplasm of retroperitoneum and peritoneum; C78.7 Secondary malignant neoplasm of liver; C78.8 Secondary malignant neoplasm of other and unspecified digestive organs	.	67	UKBB
4		C79.9 Secondary malignant neoplasm, unspecified site	.	69	UKBB
5	p.Leu550Pro	C44.1 Skin of eyelid, including canthus	Basal cell carcinoma, NOS	67.3	UKBB
5		C44.3 Skin of other and unspecified parts of face		72	UKBB
6	p.Arg552Lys	C44.9 Unspecified malignant neoplasm of skin, unspecified	.	43.2	MyCode
7	p.Tyr702His	C16.0 Cardia; C16.9 Stomach, unspecified	Adenocarcinoma, NOS	65.6	UKBB
7		C15.0 Cervical part of oesophagus;C15.9 Oesophagus, unspecified	.	66	UKBB
8	p.Tyr702His	C44.2 Skin of ear and external auricular canal	Basal cell carcinoma, NOS	72.1	UKBB

O.

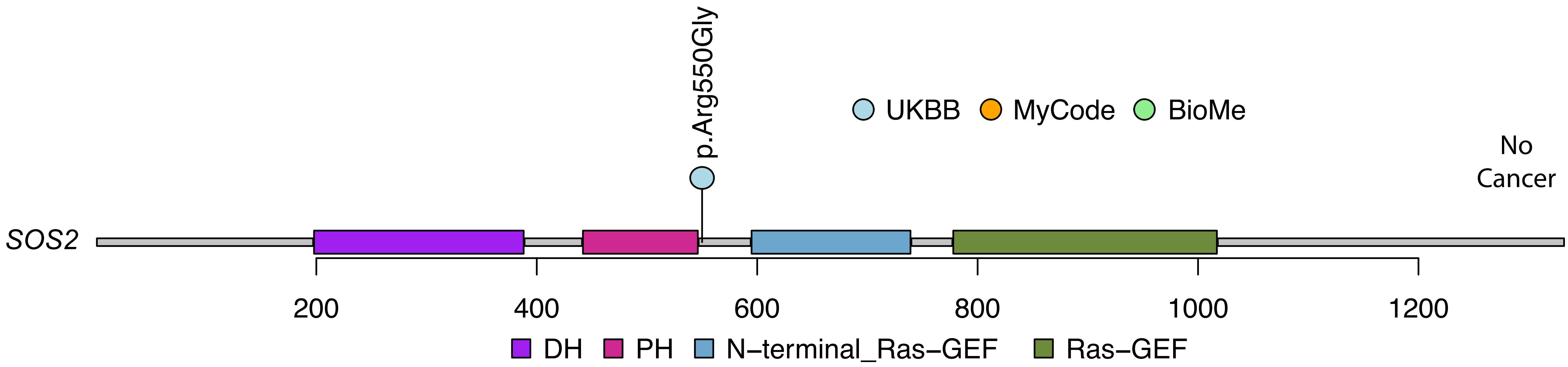
p.Arg550Gly
UKBB
MyCode
BioMe
No Cancer
SOS2
200
400
600
800
1000
1200
DH
PH
N−terminal_Ras−GEF
Ras−GEF

P.

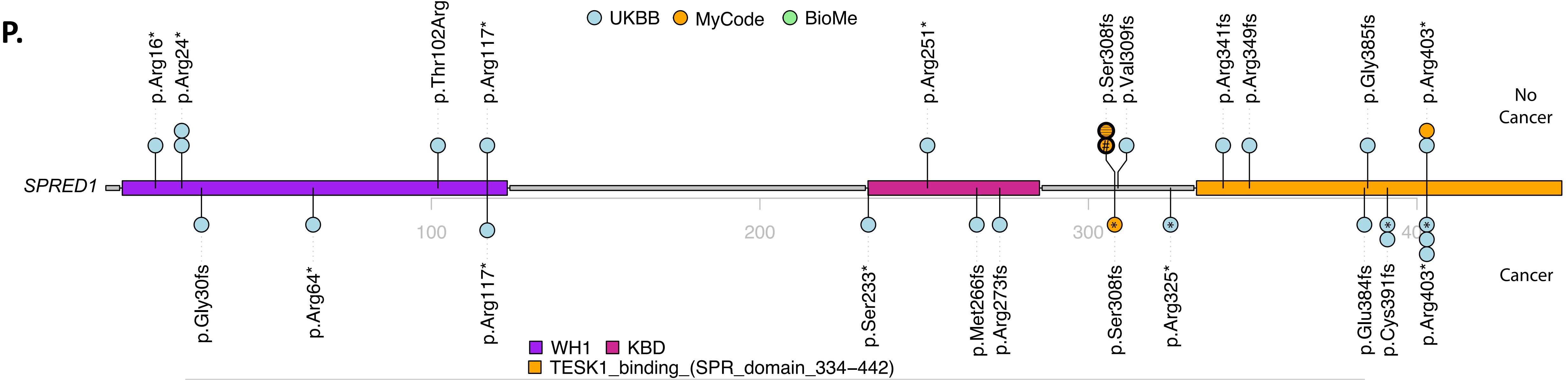

	HGVS p.	Cancer	Histology	Age at diagnosis	Database
1	p.Gly30fs	C67.9 Bladder, unspecified; C67.4 Posterior wall of bladder	Papillary trans. cell carcinoma	57.3	UKBB
2	p.Arg64*	C17.0 Duodenum	Adenocarcinoma, NOS	57.8	UKBB
2		C16.9 Stomach, unspecified	.	58	UKBB
3	p.Arg117*	C44.6 Skin of upper limb, including shoulder	.	73	UKBB
4	p.Ser233*	C44.9 Malignant neoplasm of skin, unspecified	Basal cell carcinoma, NOS	67.1	UKBB
5	p.Met266fs	C44.3 Skin of other and unspecified parts of face	Basal cell carcinoma, NOS	44	UKBB
5		C61 Malignant neoplasm of prostate	Adenocarcinoma, NOS	63.7	UKBB
6	p.Arg273fs	C44.3 Skin of other and unspecified parts of face; C44.1 Skin of eyelid, including canthus	Squamous cell carcinoma, NOS	69.6	UKBB
7	p.Ser308fs	C44.0 Basal cell carcinoma of skin of lip	.	56.4	MyCode
8	p.Arg325*	C80 Malignant neoplasm without specification of site	Adenocarcinoma, metastatic, NOS	56.7	UKBB
8		C16.9 Stomach, unspecified	.	57	UKBB
8		C78.0 Secondary malignant neoplasm of lung; C78.2 Secondary malignant neoplasm of pleura; C78.6 Secondary malignant neoplasm of retroperitoneum and peritoneum;C79.5 Secondary malignant neoplasm of bone and bone marrow	.	57	UKBB
9	p.Glu384fs	C50.9 Breast, unspecified; C50.5 Lower-outer quadrant of breast	Infiltrating duct carcinoma, NOS	58.6	UKBB
9		C80 Malignant neoplasm without specification of site	.	64	UKBB
10	p.Cys391fs	C34.9 Bronchus or lung, unspecified	Carcinoma, NOS	73	UKBB
11	p.Cys391fs	C43.4 Malignant melanoma of scalp and neck	Superficial spreading melanoma	63.8	UKBB
12	p.Arg403*	C50.8 Overlapping lesion of breast	Infiltrating duct carcinoma, NOS	63.1	UKBB
12		C50.9 Breast, unspecified	.	51	UKBB
13	p.Arg403*	C64 Malignant neoplasm of kidney, except renal pelvis	Clear cell adenocarcinoma, NOS	73.4	UKBB
13		C78.7 Secondary malignant neoplasm of liver; C79.5 Secondary malignant neoplasm of bone and bone marrow		73	UKBB
14	p.Arg403*	C44.1 Skin of eyelid, including canthus	Basal cell carcinoma, NOS	40.5	UKBB
